# Selective C-N σ Bond Cleavage in Azetidinyl Amides under Transition Metal-Free Conditions

**DOI:** 10.3390/molecules24030459

**Published:** 2019-01-28

**Authors:** Hengzhao Li, Zemin Lai, Adila Adijiang, Hongye Zhao, Jie An

**Affiliations:** College of Science, China Agricultural University, No. 2 Yuanmingyuan West Road, Beijing 100193, China; lihengzhao@cau.edu.cn (H.L.); 2016310060420@cau.edu.cn (Z.L.); 2015310060229@cau.edu.cn (A.A.); hongyezhao@cau.edu.cn (H.Z.)

**Keywords:** amides, C-N σ bond cleavage, sodium, crown ether

## Abstract

Functionalization of amide bond via the cleavage of a non-carbonyl, C-N σ bond remains under-investigated. In this work, a transition-metal-free single-electron transfer reaction has been developed for the C-N σ bond cleavage of *N*-acylazetidines using the electride derived from sodium dispersions and 15-crown-5. Of note, less strained cyclic amides and acyclic amides are stable under the reaction conditions, which features the excellent chemoselectivity of the reaction. This method is amenable to a range of unhindered and sterically encumbered azetidinyl amides.

## 1. Introduction

**Scheme 1:**
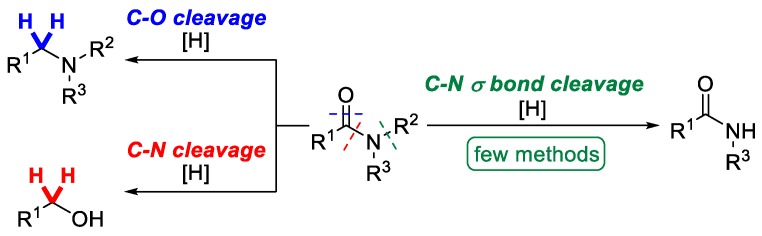
Reductive functionalization of amides.

Amide is among the most ubiquitous functional groups [1]. Although the reductive functionalization of amides has been studied extensively, the majority of strategies have focused on the amide reductions via C-O or C-N cleavage, to afford the corresponding amines or alcohols (Scheme 1) [2,3,4,5,6,7,8,9,10,11]. Only a few examples were reported for the amide bond functionalization via the selective activation of the non-carbonyl, C-N σ bond, despite its considerable potential in the synthesis of amide linkage in both chemistry and biology (Scheme 1) [12,13]. In 2005, Aube and co-workers reported a highly unusual C-N σ bond cleavage in a class of specialized bridged lactams under catalytic hydrogenation conditions [14]. The twisted amide bond is the possible reason for the high activities of the C-N σ bonds in those substrates. Recently, Szostak and co-workers have developed a more general single electron transfer (SET) method for the reductive cleavage of C-N σ bonds in both planar and pyramidalized amides, using TmI_2_-ROH reagent, which forms from a nonclassical lanthanide (II) iodide [15,16,17,18]. Given the high price of thulium, a corresponding SET protocol mediated by cheap electron donor reagents will be more desirable, which is the subject of this work.

Electrides, in which anions are electrons, are a class of useful single electron-donor reagents. Solutions of alkali metal in liquid metals [19], first described by Sir Humphry Davy in 1803 [20], are among the most common electride systems, which have found wide applications in single-electron transfer reductions, including the venerable Birch reduction [21,22,23]. To avoid the hazards that are associated with the usage of liquid ammonia, new methods for the generation of electride salts using alkali metal and crown ethers were developed. However, freshly distilled sodium, potassium mirror, or highly pyrophoric potassium–sodium alloys were required to accelerate the reaction between the alkali metal and crown ethers [24,25,26,27]. Previously, our group developed a more practical protocol for the synthesis of electride salts, using sodium dispersions and 15-crown-5 [28]. Sodium dispersion in oil is a bench-stable and commercially available reagent with a high specific surface area [28,29,30,31,32]. The derived electride has already been successfully applied in a chemoselective ammonia-free Birch reduction [28]. However, the application of such an electride in other SET reactions remains under-investigated. Herein, we report the first electride-mediated C-N σ bond cleavage reaction in pyramidalized azetidinyl amides (τ = 3.3°; *χ*_N_ = 32.5°; 4-TolC(*O*)-azetidine, Winkler−Dunitz parameters [33]), using a cheap sodium dispersion/15-crown-5 reagent system under practical conditions.

## 2. Results and Discussion

Our study began with the optimization of the reaction conditions for the C-N σ bond cleavage process in azetidinyl amides, using **1a** as a model substrate. In the previous work, we have demonstrated that **1a** can be converted into the corresponding alcohol via C-N cleavage, using Na/EtOH [5]. We hypothesized that the absence of the proton donor would suppress the amide reduction pathway, and lead to the formation of **2a** via the C-N σ bond cleavage. The initial trial of the reaction, using 5.0 equiv. of sodium dispersions in Et_2_O afforded **2a** in a moderate yield of 50% with the recovered starting material, accounting for the majority of the remaining mass balance (entry 1, Table 1). By-products derived from the amide reduction were not observed. The yield could be significantly improved by replacing Et_2_O with tetrahydrofuran (THF), a solvent with higher dielectric constant, which indicated that the reaction might go through an outer-sphere electron transfer mechanism (entry 2, Table 1). As electrides are promising electron donors for the outer electron transfer processes, the feasibility of electride derived from sodium dispersions and 15-crown-5 was investigated. When 5.0 equiv. of Na/15-crown-5 was employed, satisfactory yields of **2a** were obtained in both Et_2_O and THF (Entries 3 and 4, Table 1). Although Na/15-crown-5/*i*-PrOH is an effective system for Birch-type reductions, dearomatization was well-suppressed in the absence of a proton donor, and the reduction of the phenyl moiety in **1a** was not observed under the conditions using Na/15-crown-5 (Entries 3 and 4, Table 1). The reductive C-N σ bond cleavage is a two-electron process. However, shortening the amount of Na/15-crown-5 to 3.0 equiv. resulted in a much lower yield (entries 5 and 6, Table 1). Also, shortening the reaction time gave decreased yields (Entries 7 and 8, Table 1).

**Figure 1:**
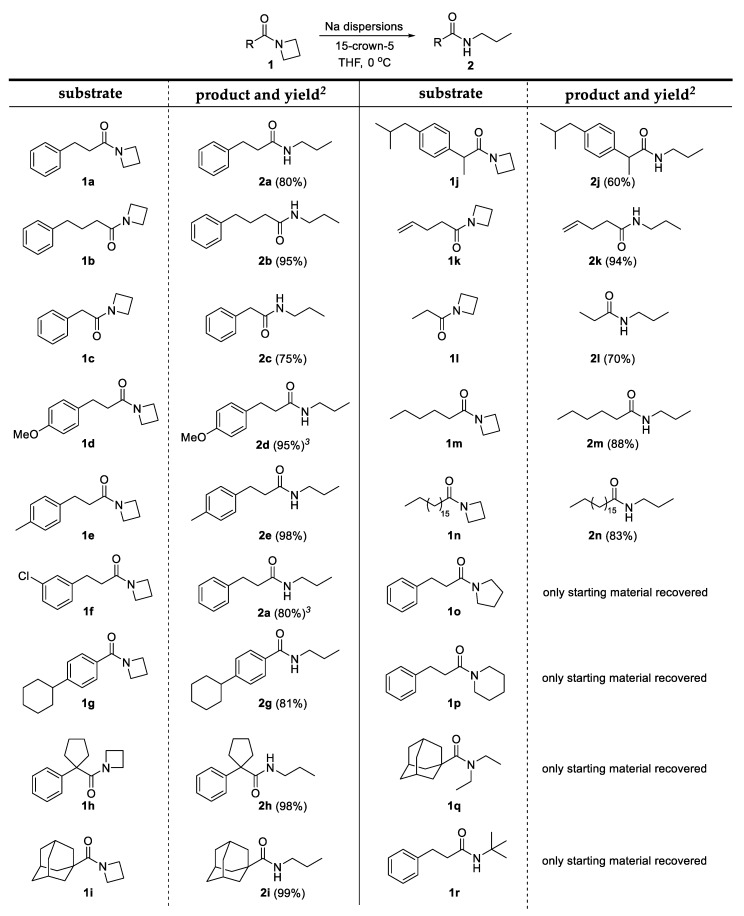
Selective C-N σ bond cleavage in azetidinyl amides by Na/15-crown-5 ^1^. ^1^ Conditions: **1** (0.50 mmol, 1.0 equiv.), Na dispersions (5.0 equiv.), 15-crown-5 (5.0 equiv.), THF (3.0 mL), 0 °C. ^2^ Isolated yield. ^3^ Na dispersions (8.0 equiv.) and 15-crown-5 (8.0 equiv.) were used.

Next, the optimized conditions (Entry 4, Table 1) were applied to the selective C-N σ bond cleavage reactions. A broad range of aliphatic and aromatic azetidinyl amides were converted into the corresponding secondary *n*-butyl amides at high yields (Figure 1). Both the unhindered (e.g., **1a**, **1g**, and **1m**) and sterically encumbered (**1h**, **1i**, and **1j**) azetidinyl amides were viable substrates for this reaction. Aromatic rings were stable under the reaction conditions. By-products derived from the Birch-type dearomatization were not detected in any of the tested substrates (**1a**–**1h**). Substrate-bearing functional groups, such as methoxy group (**1d**) and alkene group (**1k**), were also readily converted into the corresponding *n*-butyl amides without the demethylation of the methoxy group or the reduction of the alkene group. In contrast, chloride (**1f**) were fully reduced when 8.0 equiv. of Na/15-crown-5 was used, which suggested that the potential application of this protocol in dehalogenation reactions. In addition, if the reaction with **1a** was quenched by D_2_O, the corresponding deuterium labeled product, 3-phenyl-*N*-(propyl-3-*d*)propanamide, was detected, albeit in a low deuterium incorporation. Remarkably, this single electron transfer process is highly selective for azetidinyl amides. Less strained cyclic amides, such as pyrrolidinyl amide **1o** and piperidinyl amide **1p**, were very stable under the reaction conditions. Acyclic tertiary amide **1r** and secondary amide **1q** also did not undergo the cleavage reaction. Those observations suggested that the large ring strain in a four-membered ring of 25.4 kcal/mol (cf. aziridines, 27.5 kcal/mol) [33] is the possible driving force for the C-N σ bond cleavage process in azetidinyl amides.

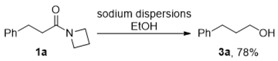
(1)

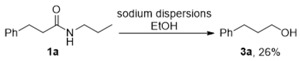
(2)

**Scheme 2:**
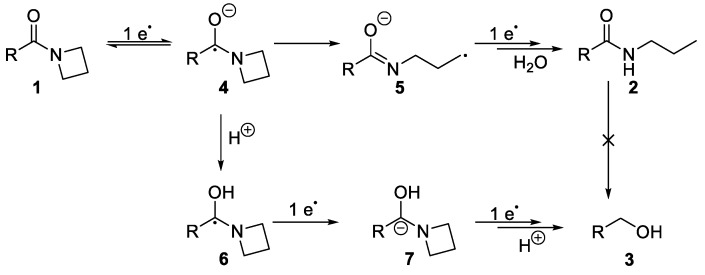
Proposed mechanism for the C-N σ bond cleavage in azetidinyl amides with Na/15-crown-5.

The key processes in this reaction involve the generation of ketyl-type radicals from a reversible electron transfer (**1**→**4**, Scheme 2) and the sequential C-N σ bond cleavage (**4**→**5**). The control reactions (Equations (1) and (2)) demonstrated that (a) in the presence of EtOH, alcohol **3** was formed as the major product; (b) it was difficult to convert secondary amide **2** to the corresponding alcohol **3** using Na/EtOH. These observations indicated that, in the presence of the proton donor, amide reduction via the C-N cleavage (**4**→**6**→**7**→**3**) was the dominant pathway. In addition, the ring-opening step (**4**→**5**) was relatively slow, so that the *n*-butyl amides **2** derived from the C-N σ bond cleavage were not detected in the reaction using Na/EtOH (Equation (1)). However, the second electron transfer (**6**→**7**) will be suppressed in the absence of a proton donor, which will alternately lead to the formation of a C-N σ bond cleavage product **2** (**4**→**5**→**2**).

## 3. Materials and Methods

### 3.1. General Information

Glassware was dried in an oven overnight before use. Thin-layer chromatography was carried out on SIL G/UV254 silica–aluminum plates, and plates were visualized using ultraviolet light (254 nm) and KMnO_4_ solution. For flash column chromatography, silica gel 60, 35–70 μm was used. NMR data was collected at 300 MHz. Data was manipulated directly from the spectrometer or via a networked PC with appropriate software. All samples were analyzed in CDCl_3_. Reference values for the residual solvent were taken as δ = 7.27 (CDCl_3_) for ^1^H-NMR; δ = 77.1 (CDCl_3_) for ^13^C-NMR. Multiplicities for coupled signals were designated using the following abbreviations: s = singlet, d = doublet, t = triplet, q = quartet, br = broad signal, and are given in Hz.

All solvents and reagents were used as supplied. Amides were prepared by the standard method [33]. **2g** and **2n** are novel compounds, and all the other compounds used in this study have been described in the literature or are commercially available. ^1^H and ^13^C NMR spectra of **2a**–**2n**, and HRMS of **2g** and **2n** are provided in the Supplementary Materials.

### 3.2. Optimization Studies (Table 1)

To a suspension of Na dispersion in oil (33.9 wt %, 1.50–2.50 mmol) in anhydrous solvent (0.5 mL), 15-crown-5 (0–2.50 mmol) was added under Ar at 0 °C and stirred vigorously for 5 min. The solution turned dark blue rapidly. A solution of substrate (0.500 mmol) in the same solvent (2.0 mL) was then added at 0 °C. After 10–120 min, the reaction was quenched by a saturated aqueous solution of NaHCO_3_ (2.0 mL), and the reaction mixture was diluted with Et_2_O (10 mL) and brine (20 mL). The aqueous layer was extracted with Et_2_O (2 × 10 mL), and the organic layers were combined, dried over Na_2_SO_4_, filtered, and concentrated. Then, the sample was analyzed by ^1^H-NMR (CDCl_3_, 300 MHz) to obtain the yield, using an internal standard (CHCl_2_CHCl_2_) and by comparison with corresponding samples.

### 3.3. General Procedure for the C-N Bond Cleavage in Azetidinyl Amides

To a suspension of Na dispersion in oil (33.9 wt %, 2.50–4.00 mmol) in anhydrous THF (0.5 mL), 15-crown-5 (2.50–4.00 mmol) was added under Ar at 0 °C, and stirred vigorously for 5 min. A solution of the substrate (0.500 mmol) in THF (2.0 mL) was then added at 0 °C. After 2 h, the reaction was quenched by a saturated aqueous solution of NaHCO_3_ (2.0 mL) and the reaction mixture was diluted with Et_2_O (10 mL) and brine (20 mL). The aqueous layer was extracted with Et_2_O (2 × 10 mL), and the organic layers were combined, dried over Na_2_SO_4_, filtered and concentrated. The crude product was purified by flash chromatography (silica, 0–50% hexane/EtOAc).

3-Phenyl-*N*-propylpropanamide (**2a**) [34]: white solid (76.5 mg, 80%). ^1^H-NMR (300 MHz, CDCl_3_) δ 7.32–7.24 (m, 2H), 7.24–7.15 (m, 3H), 5.56 (s, 1H), 3.16 (td, *J* = 7.1, 6.9 Hz, 2H), 2.96 (t, *J* = 7.7 Hz, 2H), 2.46 (t, *J* = 7.7 Hz, 2H), 1.44 (m, 2H), 0.84 (t, *J* = 7.4 Hz, 3H); ^13^C-NMR (75 MHz, CDCl_3_) δ 172.2, 140.9, 128.4, 128.3, 126.1, 41.2, 38.4, 31.8, 22.7, 11.3.

4-Phenyl-*N*-propylbutanamide (**2b**): colorless oil (97.5 mg, 95%). ^1^H-NMR (300 MHz, CDCl_3_) δ 7.33–7.23 (m, 2H), 7.23–7.13 (m, 3H), 5.49 (s, 1H), δ 3.20 (td, *J* = 7.1, 6.5 Hz, 2H), 2.65 (t, *J* = 7.5 Hz, 2H), 2.17 (t, *J* = 7.5 Hz, 2H), 1.97 (m, 2H), 1.51 (m, 2H), 0.91 (t, *J* = 7.4 Hz, 3H); ^13^C-NMR (75 MHz, CDCl_3_) δ 172.7, 141.6, 128.6, 128.4, 126. 0, 41.3, 36.0, 35.3, 27.2, 23.0, 11.4.

2-Phenyl-*N*-propylacetamide (**2c**) [35]: white solid (66.5 mg, 75%). ^1^H-NMR (300 MHz, CDCl_3_) δ 7.40–7.23 (m, 5H), 5.41 (s, 1H), 3.57 (s, 2H), δ 3.17 (td, *J* = 7.2, 6.2 Hz, 2H), 1.44 (m, 2H), 0.83 (t, *J* = 7.4 Hz, 3H); ^13^C-NMR (75 MHz, CDCl_3_) δ 171.0, 135.2, 129.5, 129.1, 127.4, 44.0, 41.4, 22.8, 11.3.

3-(4-Methoxyphenyl)-*N*-propylpropanamide (**2d**) [34]: white solid (105.1 mg, 95%). ^1^H-NMR (300 MHz, CDCl_3_) δ 7.11 (d, *J* = 8.1 Hz, 2H), 6.82 (d, *J* = 8.1 Hz, 2H), 5.53 (s, 1H), 3.77 (s, 3H), 3.16 (td, *J* = 6.7 × 2 Hz, 2H), 2.90 (t, *J* = 7.5 Hz, 2H), 2.42 (t, *J* = 7.5 Hz, 2H), 1.45 (m, 2H), 0.85 (t, *J* = 7.4 Hz, 3H); ^13^C-NMR (75 MHz, CDCl_3_) δ 172.2, 158.1, 133.0, 129.3, 114.0, 55.3, 41.2, 38.9, 31.0, 22.9, 11.3.

*N*-Propyl-3-(p-tolyl)propanamide (**2e**): white solid (100.6 mg, 98%). ^1^H-NMR (300 MHz, CDCl_3_) δ 7.18–6.99 (m, 4H), 5.72 (s, 1H), 3.16 (td, *J* = 6.5, 6.4 Hz, 2H), 2.91 (t, *J* = 7.8 Hz, 2H), 2.44 (t, *J* = 7.8 Hz, 2H), 2.30 (s, 3H), 1.45 (m, 2H), 0.85 (t, *J* = 7.4 Hz, 3H); ^13^C-NMR (75 MHz, CDCl_3_) δ 172.2, 137.9, 135.6, 129.1, 128.2, 41.2, 38.6, 31.4, 22.8, 21.0, 11.3.

3-Phenyl-*N*-propylpropanamide (**2a**) (derived from **1f**) [34]: white solid (76.5 mg, 80%).^1^H-NMR (300 MHz, CDCl_3_) δ 7.32–7.24 (m, 2H), 7.23–7.15 (m, 3H), 5.50 (s, 1H), 3.16 (td, *J* = 6.9, 6.5 Hz, 2H), 2.96 (t, *J* = 7.7 Hz, 2H), 2.46 (t, *J* = 7.7 Hz, 2H), 1.44 (m, 2H), 0.84 (t, *J* = 7.4 Hz, 3H); ^13^C-NMR (75 MHz, CDCl_3_) δ 172.1, 141.0, 128.6, 128.4, 126.3, 41.3, 38.6, 31.9, 22.9, 11.3.

4-Cyclohexyl-*N*-propylbenzamide (**2g**): white solid (99.4 mg, 81%). ^1^H-NMR (300 MHz, CDCl_3_) δ 7.73–7.66 (m, 2H), 7.26–7.21 (m, 2H), 6.32 (s, 1H), 3.39 (td, *J* = 7.4, 6.5 Hz, 2H), 2.53 (m, 1H), 1.94–1.73 (m, 5H), 1.61 (m, 2H), 1.43 – 1.22 (m, 5H), 0.96 (t, *J* = 7.4 Hz, 3H); ^13^C-NMR (75 MHz, CDCl_3_) δ 167.6, 151.6, 132.4, 127.0 (× 2), 44.5, 41.7, 34.3, 26.8, 26.1, 23.0, 11.5; HRMS (FTMS-ESI) *m*/*z*: [M + 1]^+^ calc for C_16_H_23_NO 246.1852, found 246.1849.

1-Phenyl-*N*-propylcyclopentane-1-carboxamide (**2h**): white solid (113.4 mg, 98%). ^1^H-NMR (300 MHz, CDCl_3_) δ 7.38–7.21 (m, 5H), 5.22 (s, 1H), 3.09 (td, *J* = 7.0, 5.9 Hz, 2H), 2.52–2.41 (m, 2H), 2.06–1.96 (m, 2H), 1.89–1.75 (m, 2H), 1.75–1.60 (m, 2H), 1.36 (m, 2H), 0.75 (t, *J* = 7.4 Hz, 3H); ^13^C-NMR (75 MHz, CDCl_3_) δ 176.4, 144.4, 128.6, 126.8 (× 2), 59.3, 41.4, 36.9, 24.0, 22.7, 11.1.

(3r,5r,7r)-*N*-Propyladamantane-1-carboxamide (**2i**): colorless oil (109.6 mg, 99%). ^1^H-NMR (300 MHz, CDCl_3_) δ 5.63 (s, 1H), 3.21 (td, *J* = 6.7, 6.3 Hz, 2H), 2.08–2.01 (m, 3H), 1.88–1.82 (m, 6H), 1.76–1.68 (m, 6H), 1.51 (m,2H), 0.91 (t, *J* = 7.4 Hz, 3H); ^13^C-NMR (75 MHz, CDCl_3_) δ 177.7, 41.0, 40.7, 39.4, 36.6, 28.3, 23.0, 11.4.

2-(4-Isobutylphenyl)-*N*-propylpropanamide (**2j**): colorless oil (74.2 mg, 60%). ^1^H-NMR (300 MHz, CDCl_3_) δ 7.22–7.16 (m, 2H), 7.14–7.08 (m, 2H), 5.41 (s, 1H), 3.53 (q, *J* = 7.2 Hz, 1H), 3.14 (td, *J* = 7.1, 5.9 Hz, 2H), 2.45 (d, *J* = 7.2 Hz, 2H), 1.84 (m, 1H), 1.51 (d, *J* = 7.2 Hz, 3H), 1.41 (m, 2H), 0.90 (d, *J* = 6.6 Hz, 6H), 0.80 (t, *J* = 7.4 Hz, 3H); ^13^C-NMR (75 MHz, CDCl_3_) δ 174.5, 140.7, 138.8, 129.6, 127.4, 46.8, 45.1, 41.3, 30.2, 22.8, 22.4, 18.5, 11.2.

*N*-Propylpent-4-enamide (**2k**): colorless oil (66.4 mg, 94%). ^1^H-NMR (300 MHz, CDCl_3_) δ 5.82 (t, *J* = 8.7 Hz, 1H), 5.69 (s, 1H), 5.04 (dd, *J* = 18.9, 13.6 Hz, 2H), 3.22 (d, *J* = 6.8 Hz, 2H), 2.33 (dt, *J* = 35.4, 7.5 Hz, 4H), 1.52 (dd, *J* = 14.5, 7.3 Hz, 2H), 0.92 (t, *J* = 7.2 Hz, 3H); ^13^C-NMR (75 MHz, CDCl_3_) δ 172.3, 137.2, 115.5, 41.3 36.0, 29.8, 22.9, 11.4.

*N*-Propylpropionamide (**2l**) [36]: colorless oil (40.3 mg, 70%). ^1^H-NMR (300 MHz, CDCl_3_) δ 5.44 (s, 1H), 3.22 (td, *J* = 7.2, 6.5 Hz, 2H), 2.20 (q, *J* = 7.6 Hz, 2H), 1.52 (m, 2H), 1.16 (t, *J* = 7.6 Hz, 3H), 0.93 (t, *J* = 7.6 Hz, 3H); ^13^C-NMR (75 MHz, CDCl_3_) δ 173.7, 41.3, 29.9, 23.0, 11.4, 10.0.

*N*-Propylhexanamide (**2m**): colorless oil (69.2 mg, 88%). ^1^H-NMR (300 MHz, CDCl_3_) δ 5.64 (s, 1H), 3.21 (td, *J* = 7.2, 6.1 Hz, 2H), 2.16 (t, *J* = 7.8 Hz, 2H), 1.63 (m, 2H), 1.52 (m, 2H), 1.36–1.26 (m, 4H), 0.96–0.85 (m, 6H); ^13^C-NMR (75 MHz, CDCl_3_) δ 173.2, 41.2, 36.9, 31.5, 25.6, 23.0, 22.5, 14.0, 11.4.

*N*-Propylstearamide (**2n**): white solid (135.1 mg, 83%). ^1^H-NMR (300 MHz, CDCl_3_) δ 5.63 (s, 1H), 3.21 (td, *J* = 6.9, 6.5 Hz, 2H), 2.16 (t, *J* = 7.6 Hz, 2H), 1.62 (m, 2H), 1.52 (m, 2H), 1.37–1.20 (m, 28H), 0.96–0.82 (m, 6H); ^13^C-NMR (75 MHz, CDCl_3_) δ 173.2, 41.2, 37.0, 32.0, 29.8 (× 5), 29.7 (× 3), 29.6, 29.4 (× 3), 25.9, 23.0, 22.7, 14.2, 11.4; HRMS (FTMS-ESI) *m*/*z*: [M + 1]^+^ calc for C_21_H_43_NO 326.3417, found 326.3408.

## 4. Conclusions

In summary, a transition metal-free method for the challenging C-N σ bond cleavage in azetidinyl amides has been developed, using sodium dispersions and 15-crown-5. This practical reaction requires only inexpensive air- and moisture-stable reagents. High yields were obtained across a broad range of aliphatic and aromatic azetidinyl amides. More importantly, full chemoselectivity over the reductive C-N σ bond cleavage of less strained cyclic amides and acyclic amides was achieved. This work represents the first application of an electride in the C-N σ bond cleavage in pyramidalized amides. The further application of the electride derived from sodium dispersions and crown ethers in new SET reactions will be the subject of our future research.

## Figures and Tables

**Table 1:**
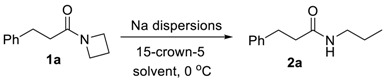
Optimization of selective C-N σ bond cleavage in azetidinyl amides by Na/15-crown-5 ^1^.

**Entry**	Sodium Dispersion ^1^ (Equiv)	15-crown-5 (Equiv)	Time	Solvent	Yield (%) ^2^
1	5.0	0	2.0 h	Et_2_O	50
2	5.0	0	2.0 h	THF	67
3	5.0	5.0	2.0 h	Et_2_O	79
4	5.0	5.0	2.0 h	THF	80
5	4.5	4.5	2.0 h	Et_2_O	65
6	3.0	3.0	2.0 h	Et_2_O	56
7	5.0	5.0	1.0 h	THF	71
8	5.0	5.0	10 min	THF	47

THF: tetrahydrofuran. ^1^ Sodium dispersions in oil, 33.9 wt %, particle size 5–10 µm. ^2^ Determined by ^1^H-NMR.

## Supplementary Materials

The following are available online at http://www.mdpi.com/1420-3049/24/3/459/s1, ^1^H and ^13^C-NMR spectra of **2a**–**2n**, and HRMS of **2g** and **2n**.

## References

[1] Greenberg A., Breneman C.M., Liebman J.F. (2000). The Amide Linkage: Structural Significance in Chemistry, Biochemistry and Materials Science.

[2] Volkov A., Tinnis F., Slagbrand T., Trillo P., Adolfsson H. (2016). Chemoselective reduction of carboxamides. Chem. Soc. Rev..

[3] Dub P.A., Ikariya T. (2012). Catalytic reductive transformations of carboxylic and carbonic acid derivatives using molecular hydrogen. ACS Catal..

[4] Addis D., Das S., Junge K., Beller M. (2011). Selective reduction of carboxylic acid derivatives by catalytic hydrosilylation. Angew. Chemie Int. Ed..

[5] Zhang B., Li H., Ding Y., Yan Y., An J. (2018). Reduction and reductive deuteration of tertiary amides mediated by sodium dispersions with distinct proton donor-dependent chemoselectivity. J. Org. Chem..

[6] Rasu L., John J.M., Stephenson E., Endean R., Kalapugama S., Clément R., Bergens S.H. (2017). Highly enantioselective hydrogenation of amides via dynamic kinetic resolution under low pressure and room temperature. J. Am. Chem. Soc..

[7] Tinnis F., Volkov A., Slagbrand T., Adolfsson H. (2016). Chemoselective reduction of tertiary amides under thermal control: formation of either aldehydes or amines. Angew. Chemie Int. Ed..

[8] Mukherjee D., Shirase S., Mashima K., Okuda J. (2016). Chemoselective reduction of tertiary amides to amines catalyzed by triphenylborane. Angew. Chemie Int. Ed..

[9] Cabrero-Antonino J.R., Alberico E., Drexler H.-J., Baumann W., Junge K., Junge H., Beller M. (2016). Efficient base-free hydrogenation of amides to alcohols and amines catalyzed by well-defined pincer imidazolyl–ruthenium complexes. ACS Catal..

[10] Szostak M., Spain M., Eberhart A.J., Procter D.J. (2014). Highly chemoselective reduction of amides (primary, secondary, tertiary) to alcohols using SmI_2_ /amine/H_2_O under mild conditions. J. Am. Chem. Soc..

[11] Das S., Wendt B., Möller K., Junge K., Beller M. (2012). Two iron catalysts are better than one: A general and convenient reduction of aromatic and aliphatic primary amides. Angew. Chemie Int. Ed..

[12] Hu F., Nareddy P., Lalancette R., Jordan F., Szostak M. (2017). σ N-C Bond Difunctionalization in Bridged Twisted Amides: Sew-and-Cut Activation Approach to Functionalized Isoquinolines. Org. Lett..

[13] Hu F., Lalancette R., Szostak M. (2016). Structural Characterization of N-Alkylated Twisted Amides: Consequences for Amide Bond Resonance and N-C Cleavage. Angew. Chemie Int. Ed..

[14] Lei Y., Wrobleski A.D., Golden J.E., Powell D.R., Aubé J. (2005). Facile C-N cleavage in a series of bridged lactams. J. Am. Chem. Soc..

[15] Szostak M., Spain M., Procter D.J. (2013). Uncovering the importance of proton donors in TmI_2_-promoted electron transfer: facile C−N bond cleavage in unactivated amides. Angew. Chemie Int. Ed..

[16] Shi S., Szostak M. (2017). Synthesis of nitrogen heterocycles using samarium (II) iodide. Molecules.

[17] Shi S., Szostak R., Szostak M. (2016). Proton-coupled electron transfer in the reduction of carbonyls using SmI_2_–H_2_O: implications for the reductive coupling of acyl-type ketyl radicals with SmI_2_–H_2_O. Org. Biomol. Chem..

[18] Shi S., Szostak M. (2015). Aminoketyl radicals in organic synthesis: Stereoselective cyclization of five- and six-membered cyclic imides to 2-azabicycles using SmI_2_–H_2_O. Org. Lett..

[19] Thompson J.C. (1976). Electrons in Liquid Ammonia.

[20] Thomas S.J.M., Edwards P.P., Kuznetsov V.L. (2008). Sir Humphry Davy: boundless Chemist, physicist, poet and man of action. Chem. Phys. Chem..

[21] Rabideau P.W., Marcinow Z. (1992). The Birch reduction of aromatic compounds. Organic Reactions.

[22] Zimmerman H.E. (2012). A mechanistic analysis of the Birch reduction. Acc. Chem. Res..

[23] Cossy J., Gille B., Bellosta V. (1998). Facile synthesis of spirocyclic systems through the intramolecular addition of ketyl radicals via the sodium/ammonia reduction of δ,ε-unsaturated carboxylic esters. J. Org. Chem..

[24] Dye J.L. (2003). Electrons as anions. Science.

[25] Dye J.L. (1990). Electrides: ionic salts with electrons as the anions. Science.

[26] Dye J.L. (1979). Compounds of alkali metal snions. Angew. Chemie Int. Ed. English.

[27] Jedliński Z. (1998). Novel electron-transfer reactions mediated by alkali metals complexed by macrocyclic ligand. Acc. Chem. Res..

[28] Lei P., Ding Y., Zhang X., Adijiang A., Li H., Ling Y., An J. (2018). A practical and chemoselective ammonia-free Birch reduction. Org. Lett..

[29] Ding Y., Luo S., Adijiang A., Zhao H., An J. (2018). Reductive deuteration of nitriles: the synthesis of α,α-dideuterio amines by sodium mediated electron transfer reactions. J. Org. Chem..

[30] Han M., Ding Y., Yan Y., Li H., Luo S., Adijiang A., Ling Y., An J. (2018). Transition-metal-free, selective reductive deuteration of terminal alkynes with sodium dispersions and EtOD-*d*_1_. Org. Lett..

[31] Li H., Zhang B., Dong Y., Liu T., Zhang Y., Nie H., Yang R., Ma X., Ling Y., An J. (2017). A selective and cost-effective method for the reductive deuteration of activated alkenes. Tetrahedron Lett..

[32] Han M., Ma X., Yao S., Ding Y., Yan Z., Adijiang A., Wu Y., Li H., Zhang Y., Lei P. (2017). Development of a modified Bouveault–Blanc reduction for the selective synthesis of α,α-dideuterio alcohols. J. Org. Chem..

[33] Liu C., Achtenhagen M., Szostak M. (2016). Chemoselective ketone synthesis by the addition of organometallics to *N*-Acylazetidines. Org. Lett..

[34] Štefane B., Polanc S. (2009). hydrogenation of BF_2_ complexes with 1,3-dicarbonyl ligands. Tetrahedron.

[35] Ignatenko V.A., Deligonul N., Viswanathan R. (2010). Branch-Selective Synthesis of Oxindole and Indene Scaffolds: Transition Metal-Controlled Intramolecular Aryl Amidation Leading to C3 Reverse-Prenylated Oxindoles. Org. Lett..

[36] Gajda T., Zwierzak A. (1981). Phase-transfer-catalysed *N*-alkylation of carboxamides and sulfonamides. Synthesis.

