# Supplementary material for: Selective C-N σ Bond Cleavage in Azetidinyl Amides under Transition Metal-Free Conditions
Source: Molecules. 2019 Jan 28;24(3):459. doi: 10.3390/molecules24030459 (PMC6384560; doi:10.3390/molecules24030459)
Supplement: Supplementary file 1 [file molecules-24-00459-s001.pdf]

molecules

Supplementary Materials

Selective C-N σ Bond Cleavage in Azetidinyl Amides under Transition Metal Free Conditions

Hengzhao Li, Zemin Lai, Adila Adijiang, Hongye Zhao and Jie An*

College of Science, China Agricultural University, No. 2 Yuanmingyuan West Road, Beijing 100193, China

Copies of ^{1}H-NMR, ^{13}C-NMR 2a–2n and HRMS Spectrum of 2g and 2n

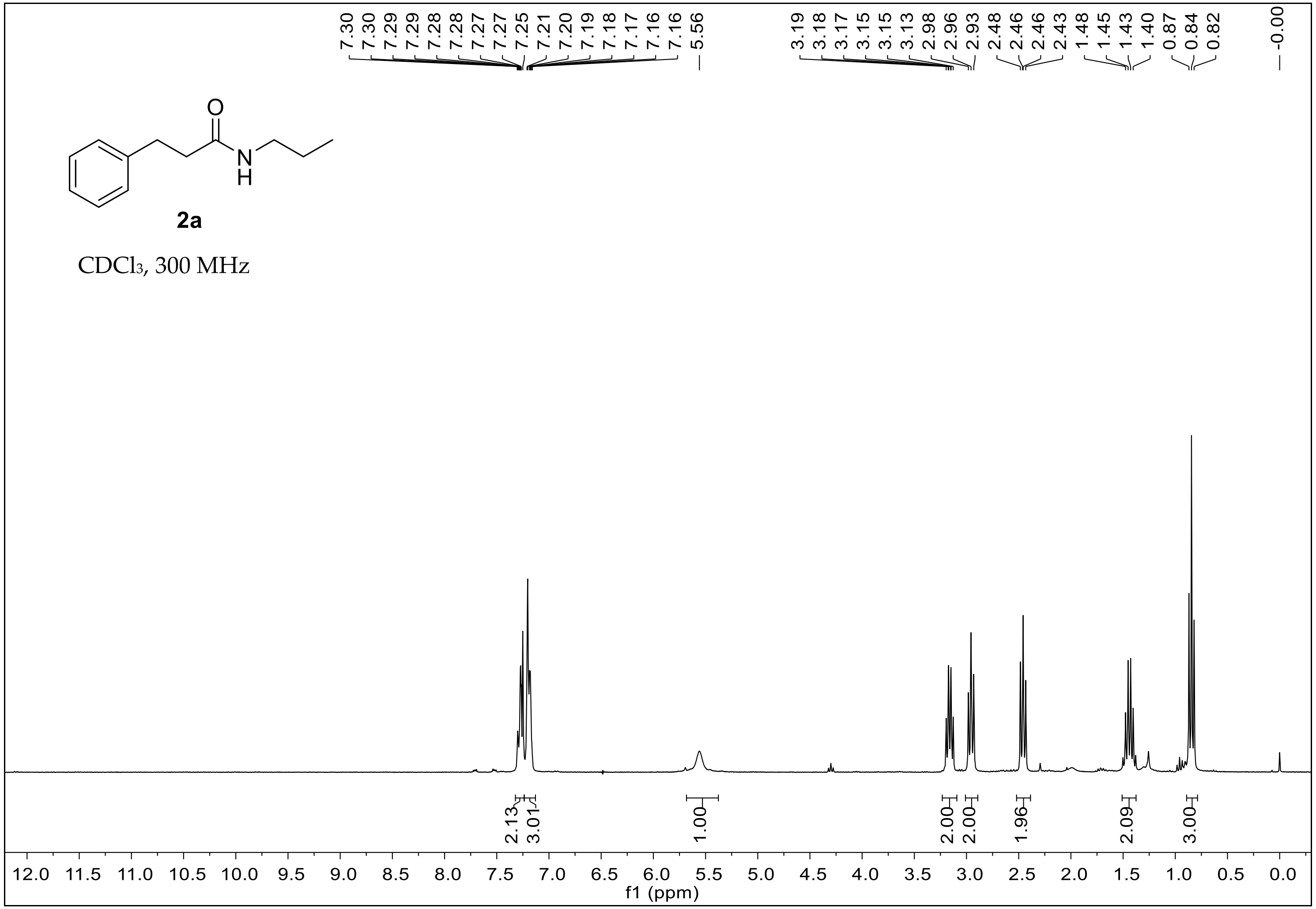

Figure S1. ^{1}H-NMR of 3-Phenyl-*N*-propylpropanamide (**2a**).

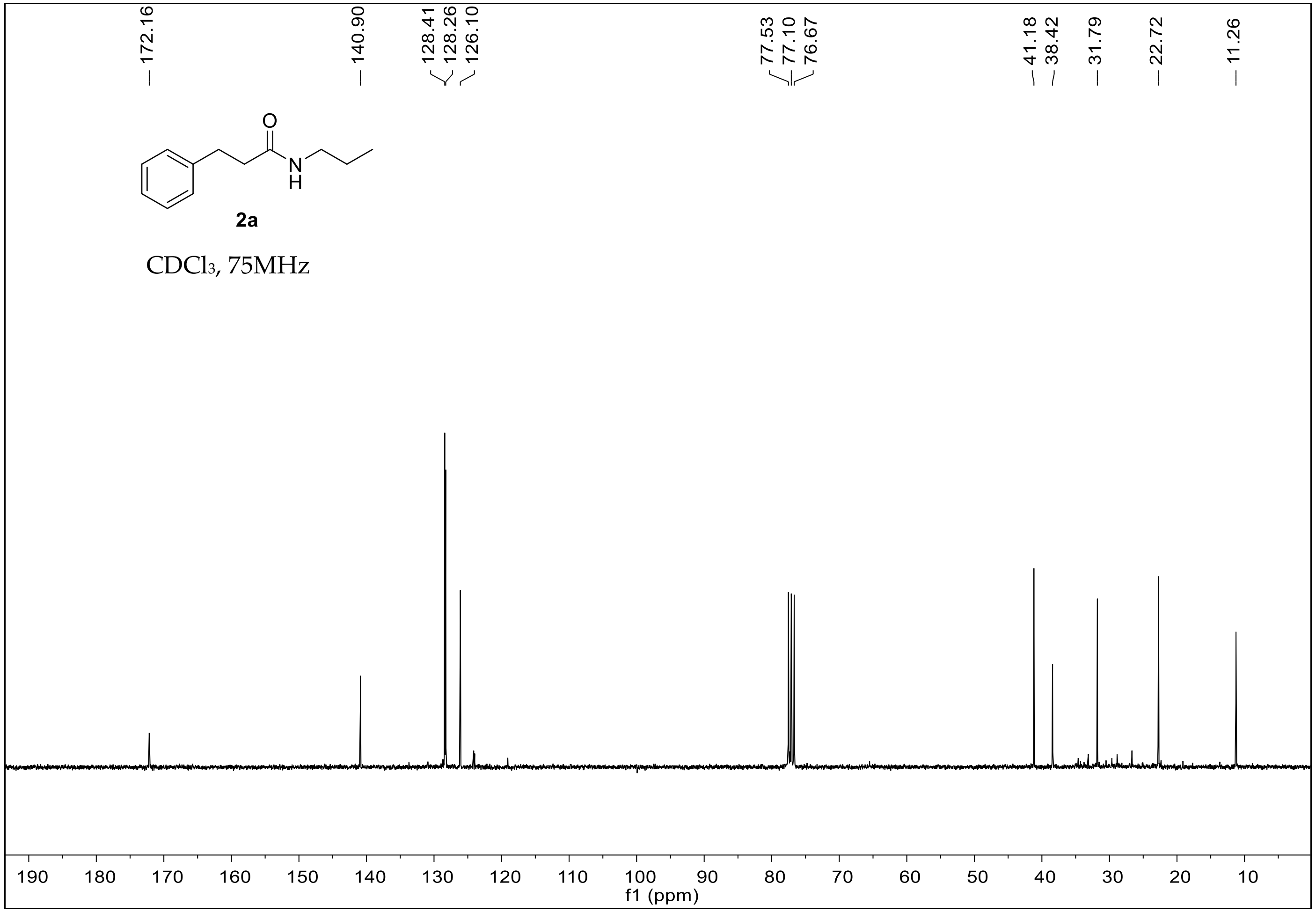

Figure S2. ^{13}C-NMR of 3-Phenyl-*N*-propylpropanamide (**2a**).

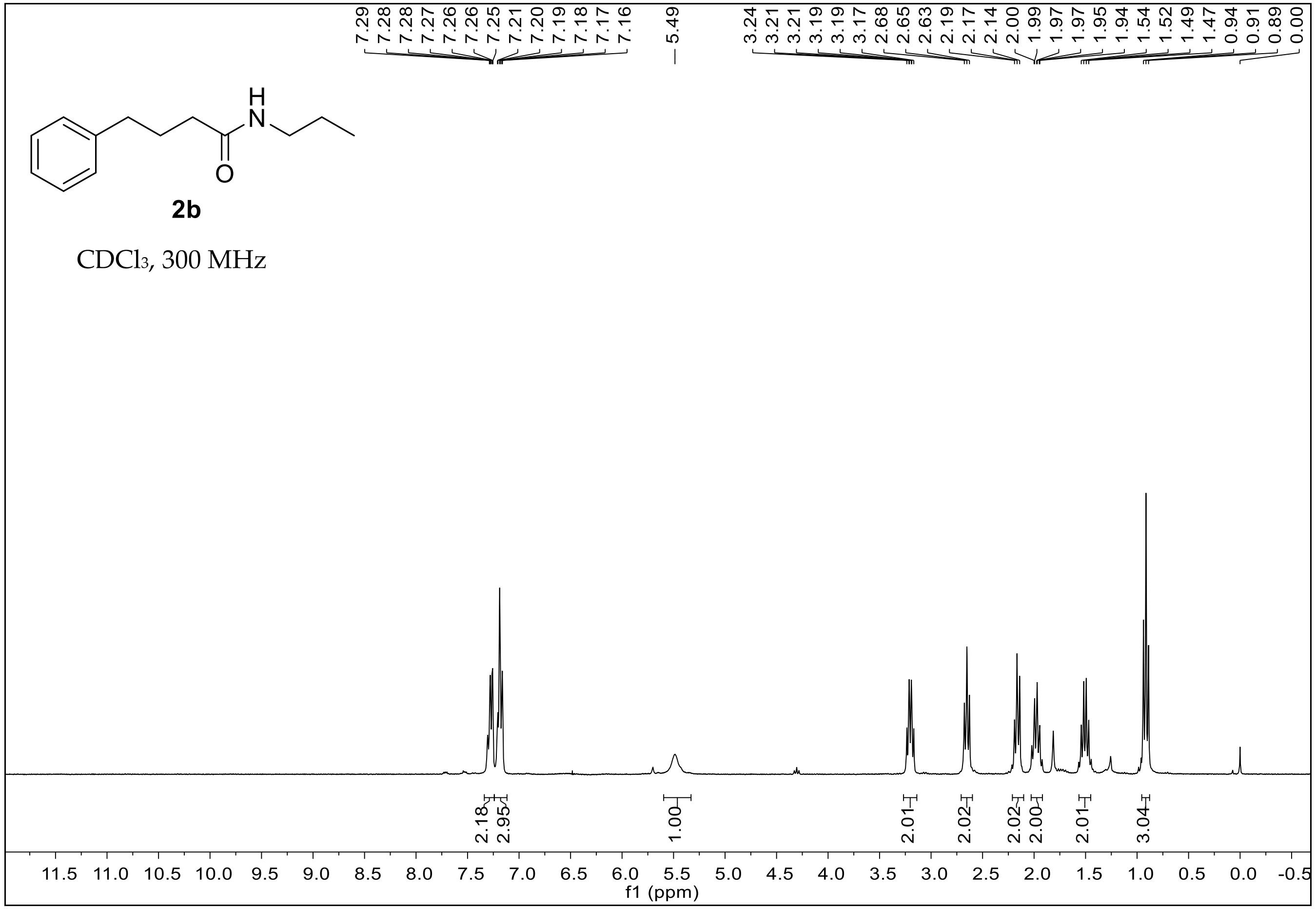

Figure S3. ^{1}HNMR of 4-Phenyl-*N*-propylbutanamide (**2b**).

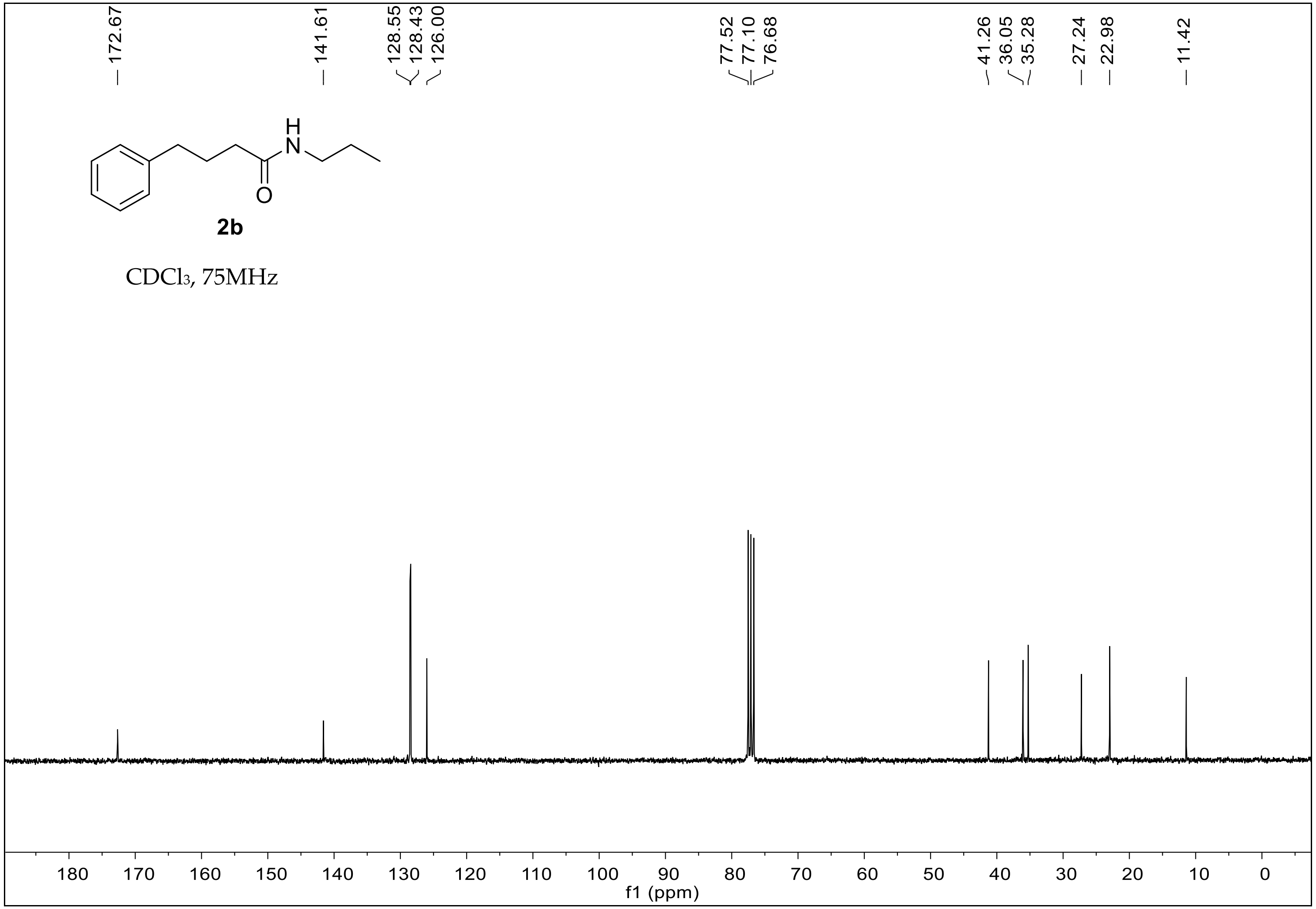

Figure S4. ^{13}C-NMR of 4-Phenyl-*N*-propylbutanamide (**2b**).

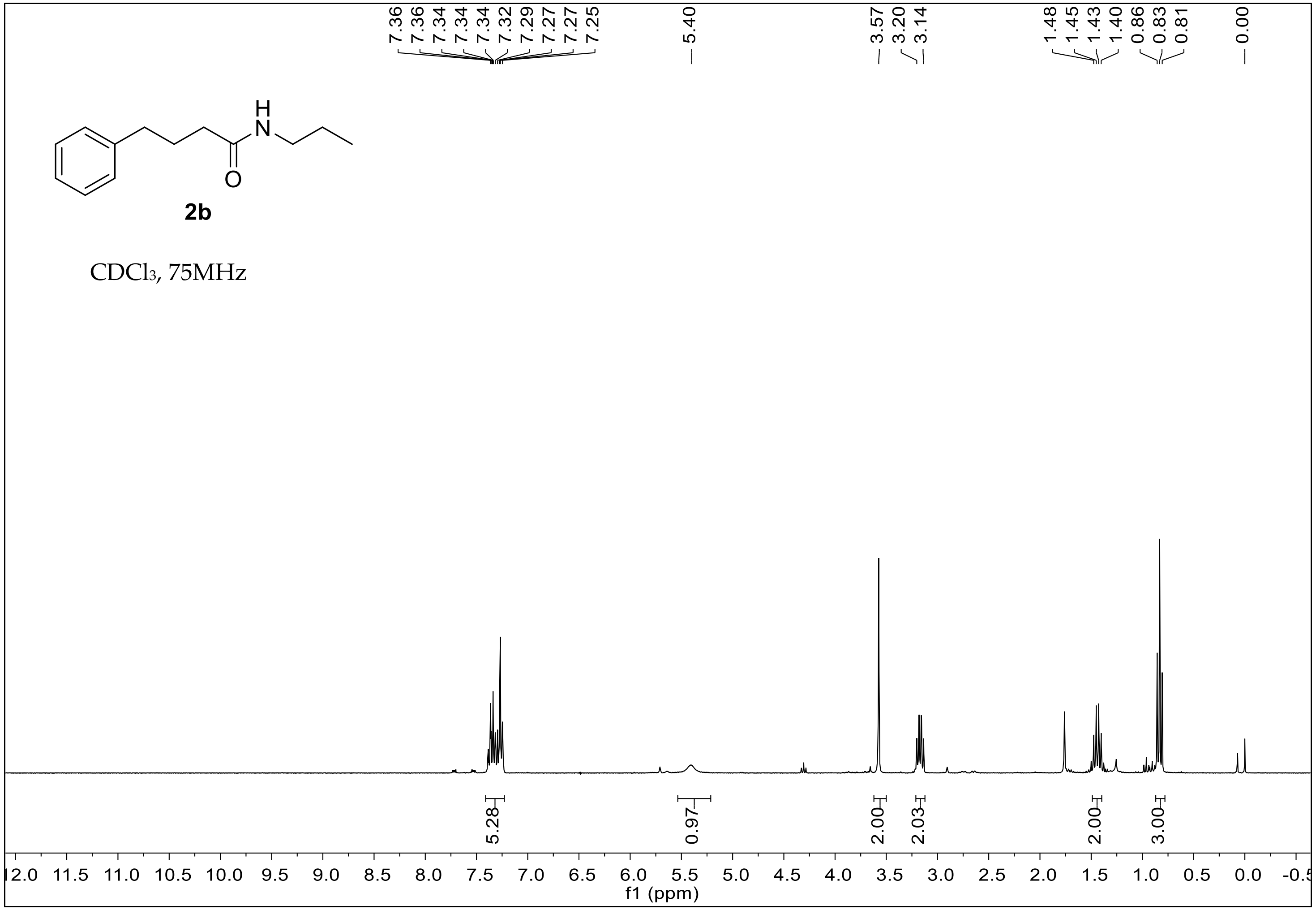

Figure S5. ^{1}H-NMR of 2-Phenyl-*N*-propylacetamide (**2c**).

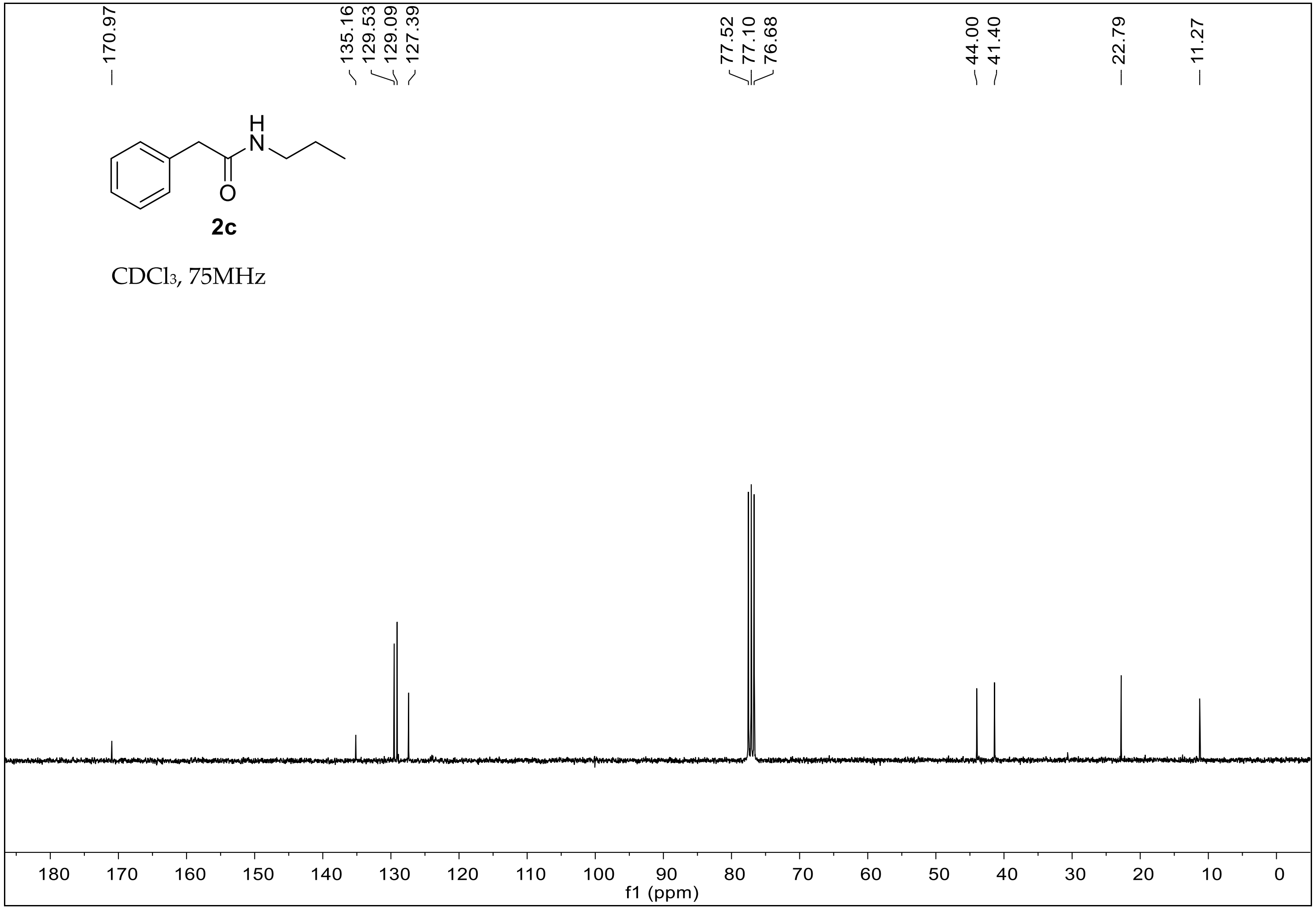

Figure S6. ^{13}C-NMR of 2-Phenyl-*N*-propylacetamide (**2c**).

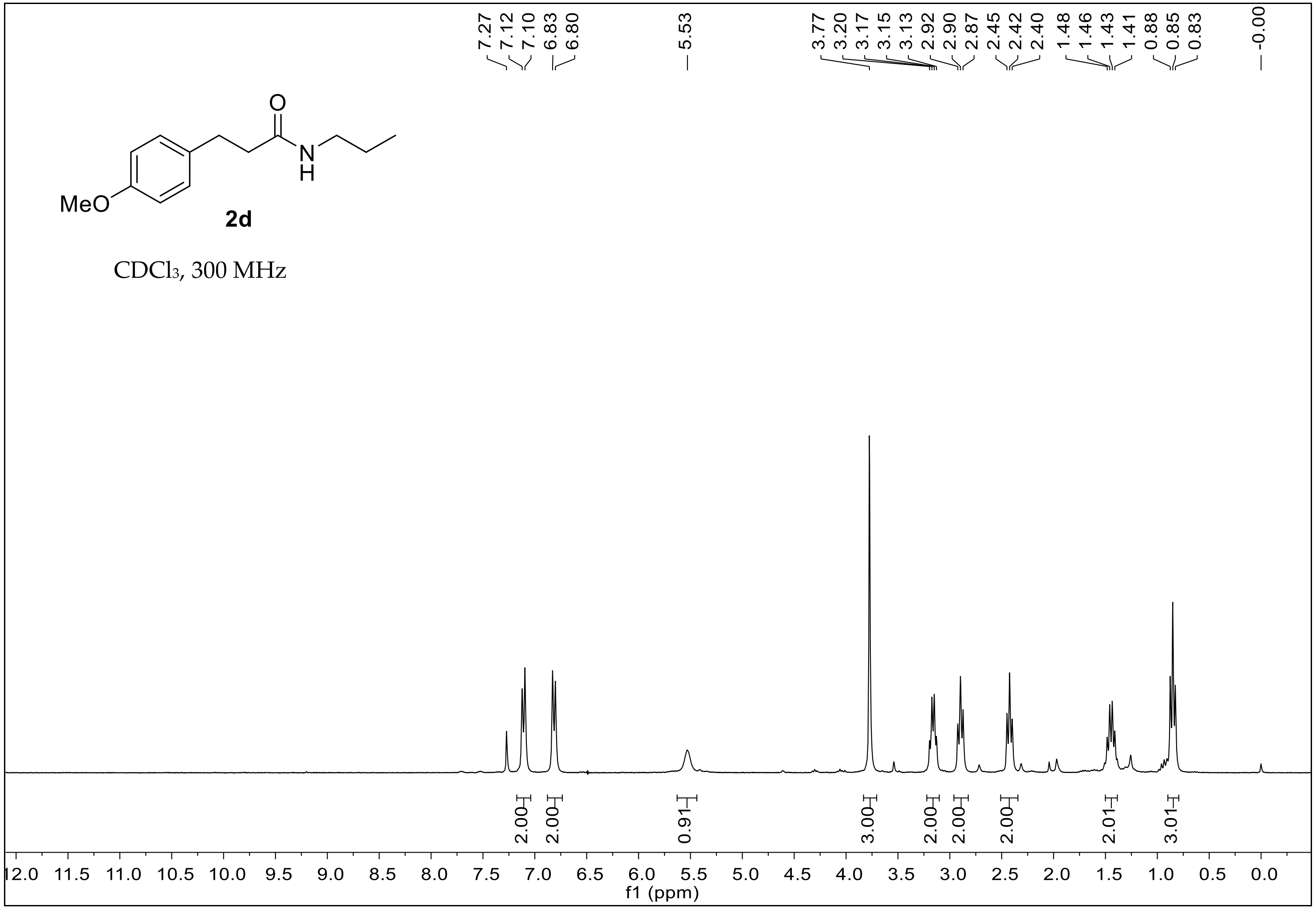

Figure S7. ^{1}H-NMR of 3-(4-Methoxyphenyl)-*N*-propylpropanamide (**2d**).

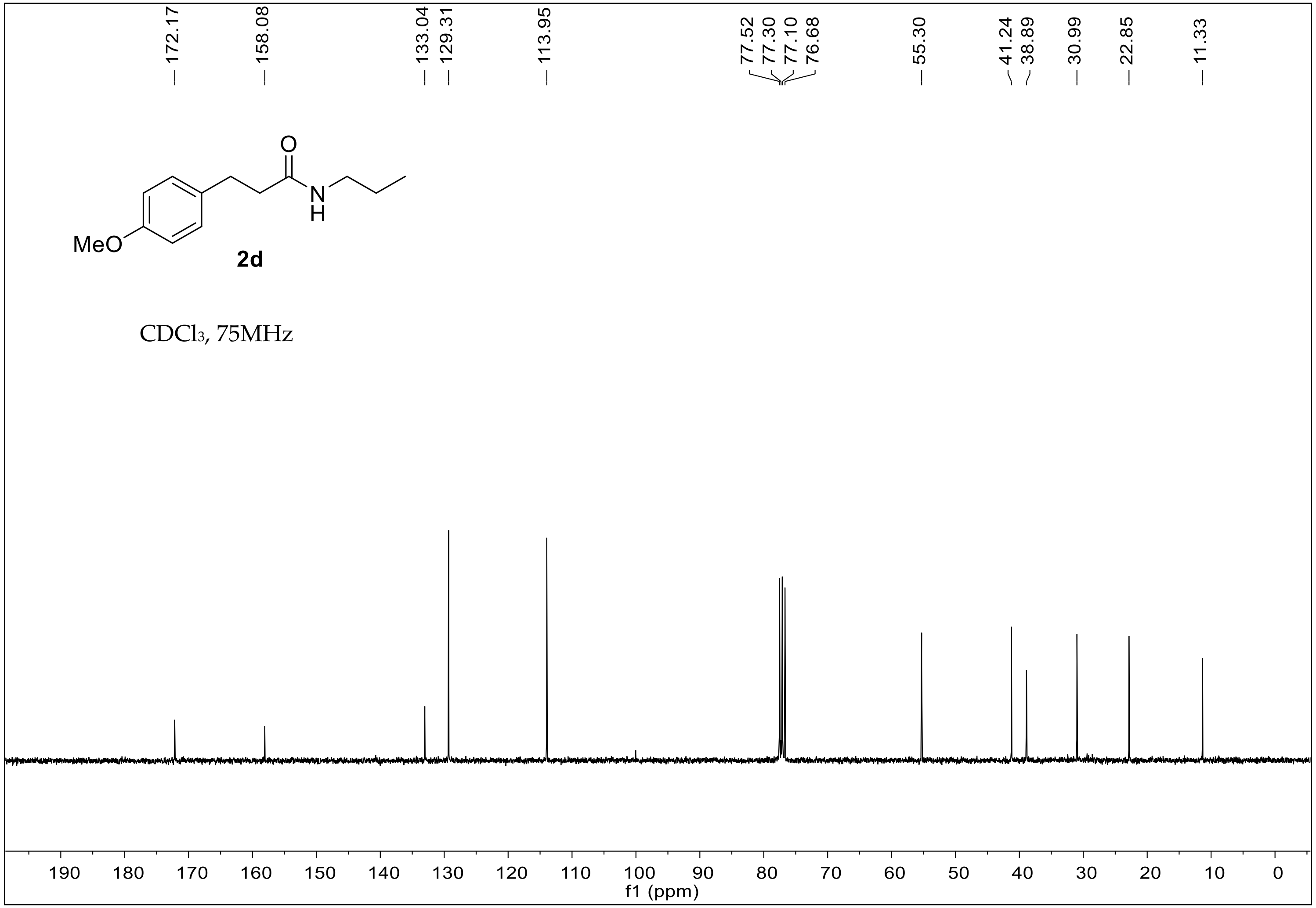

Figure S8. ^{13}C-NMR of 3-(4-Methoxyphenyl)-*N*-propylpropanamide (**2d**).

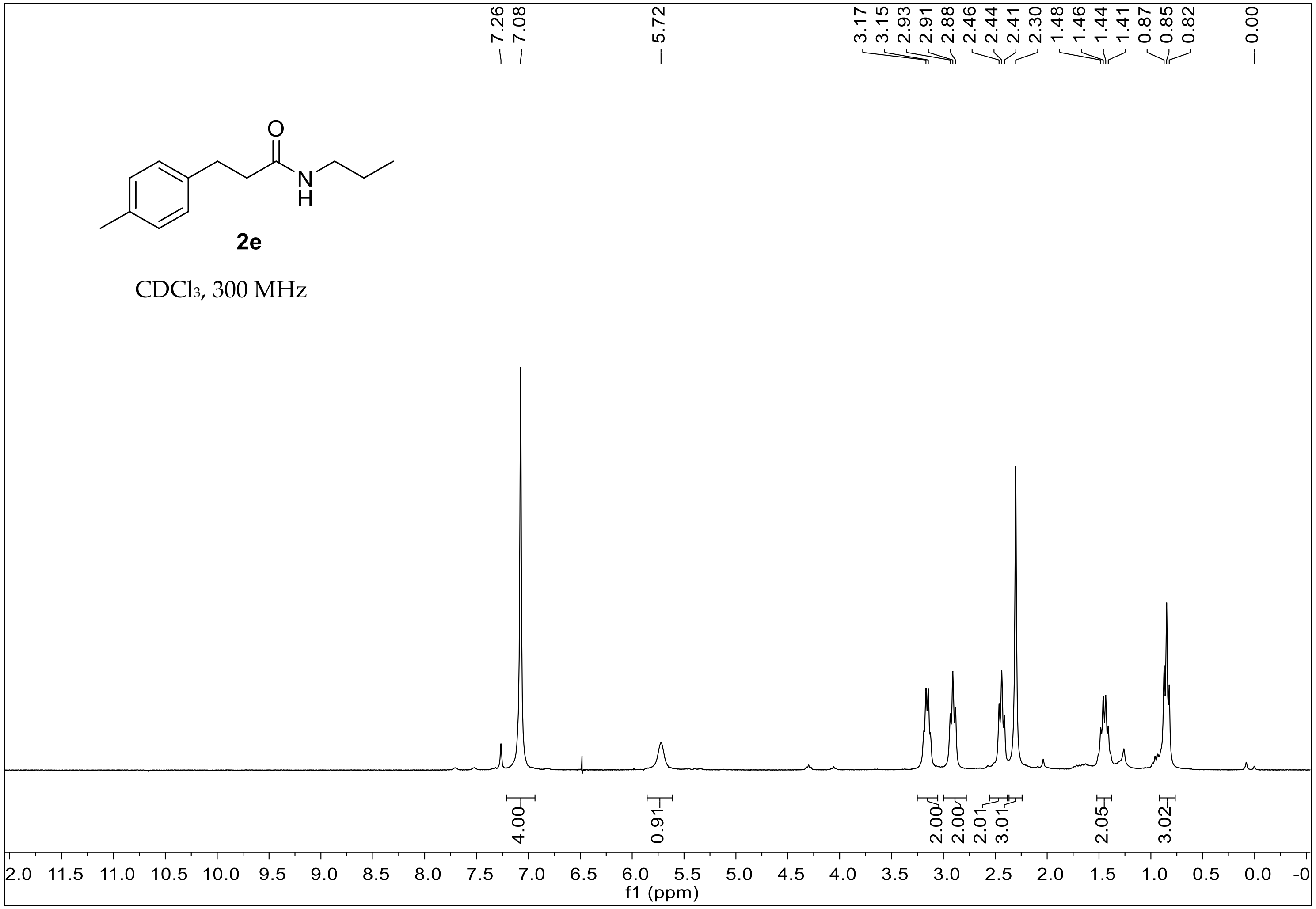

Figure S9. ^{1}H-NMR of *N*-Propyl-3-(p-tolyl)propanamide (**2e**).

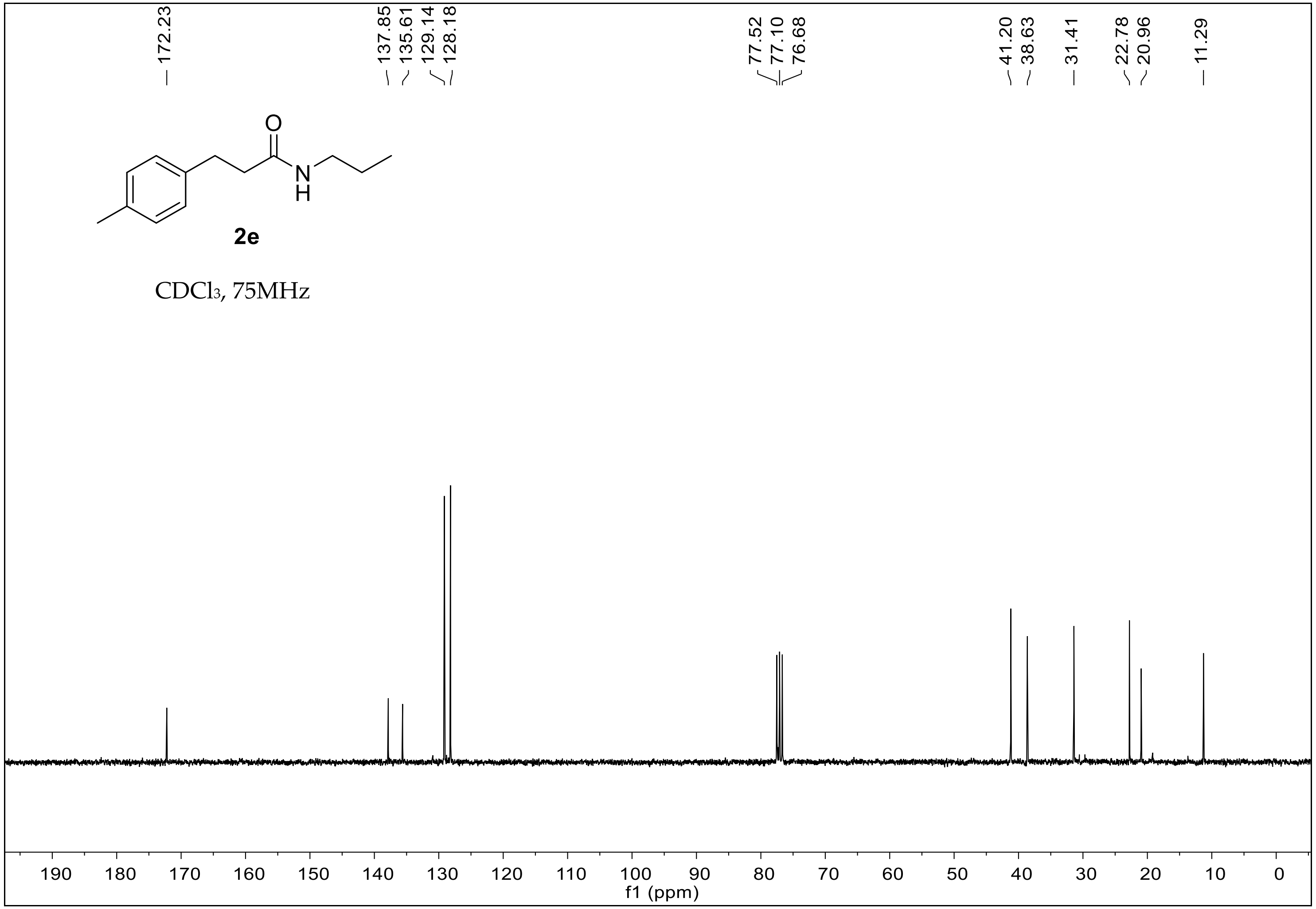

Figure S10. ^{13}C-NMR of *N*-Propyl-3-(*p*-tolyl)propanamide (**2e**).

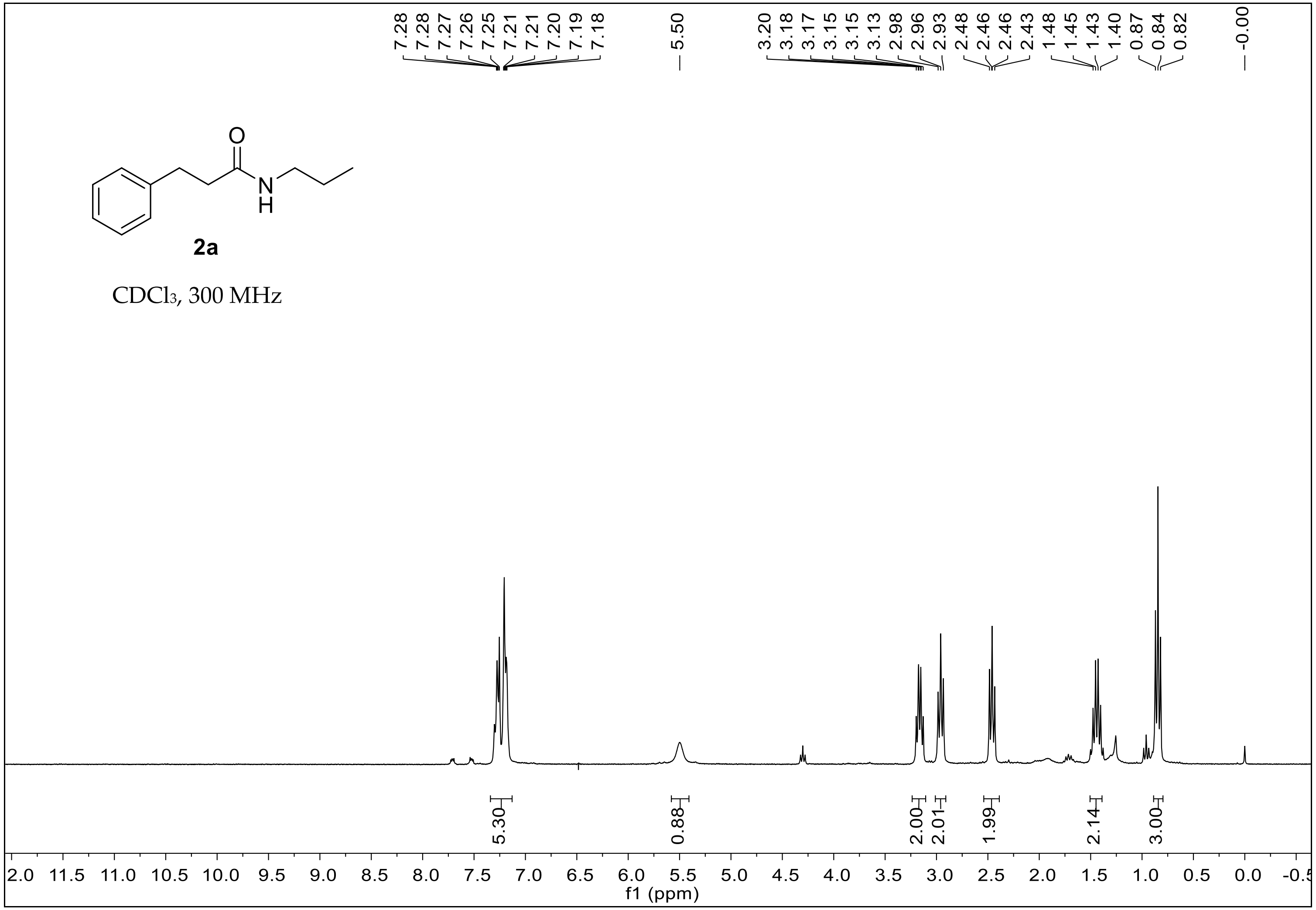

Figure S11. ^{1}H-NMR of 3-Phenyl-*N*-propylpropanamide ((**2a**) (derived from **1f**)).

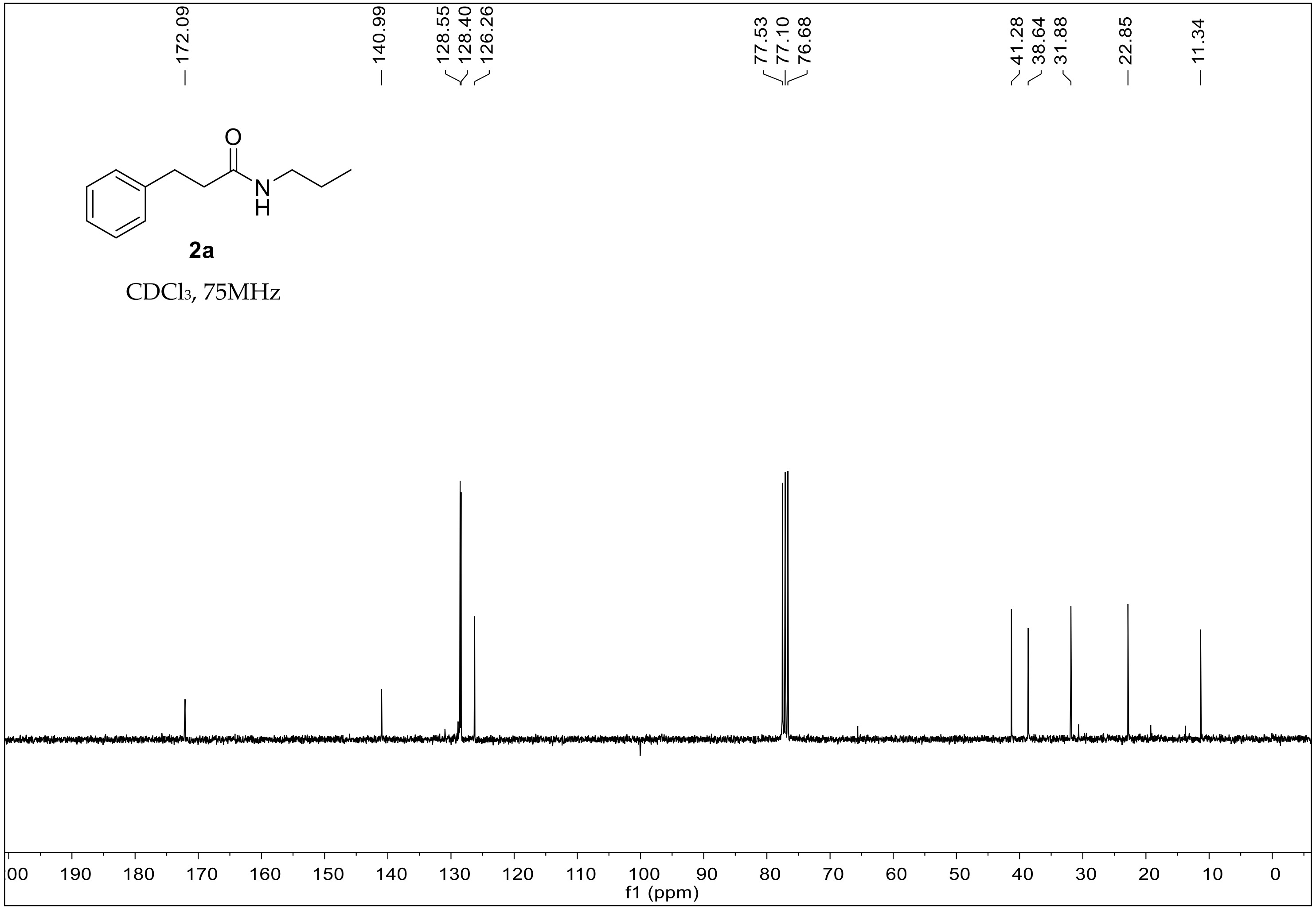

Figure S12. ^{13}C-NMR of 3-Phenyl-*N*-propylpropanamide ((**2a**) (derived from **1f**)).

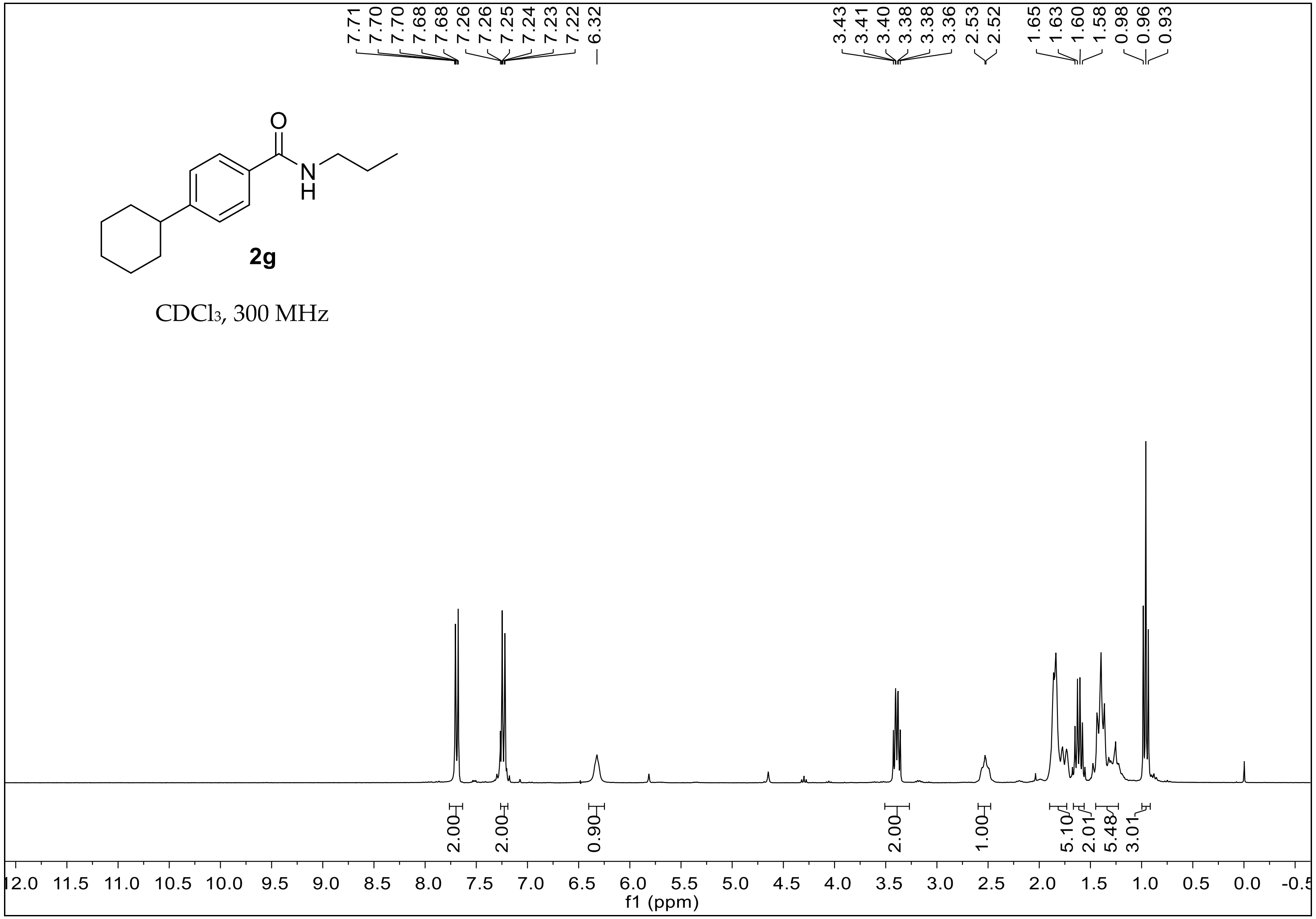

Figure S13. ¹H-NMR of 4-Cyclohexyl-*N*-propylbenzamide (**2g**).

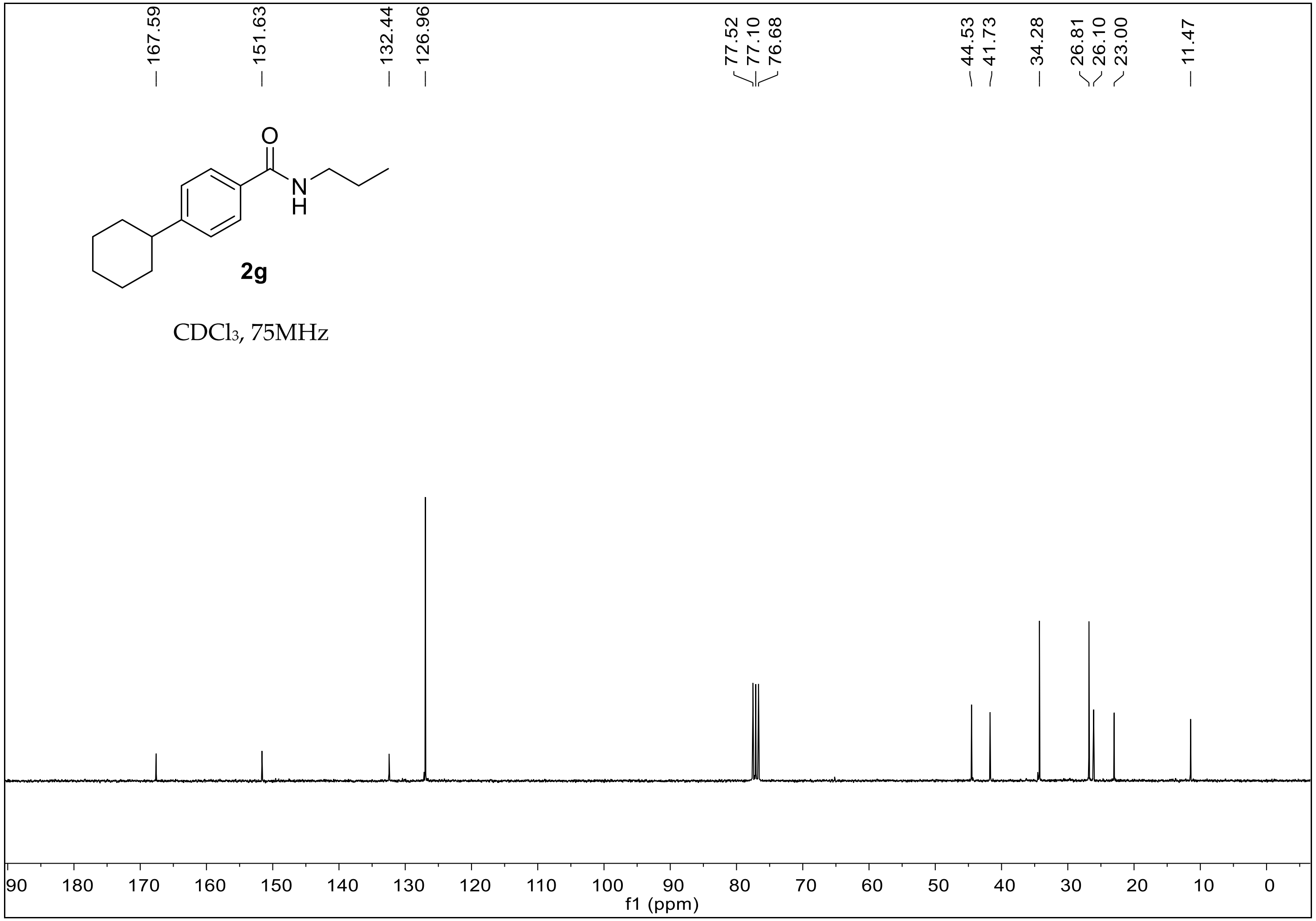

Figure S14. ^{13}C-NMR of 4-Cyclohexyl-*N*-propylbenzamide (**2g**).

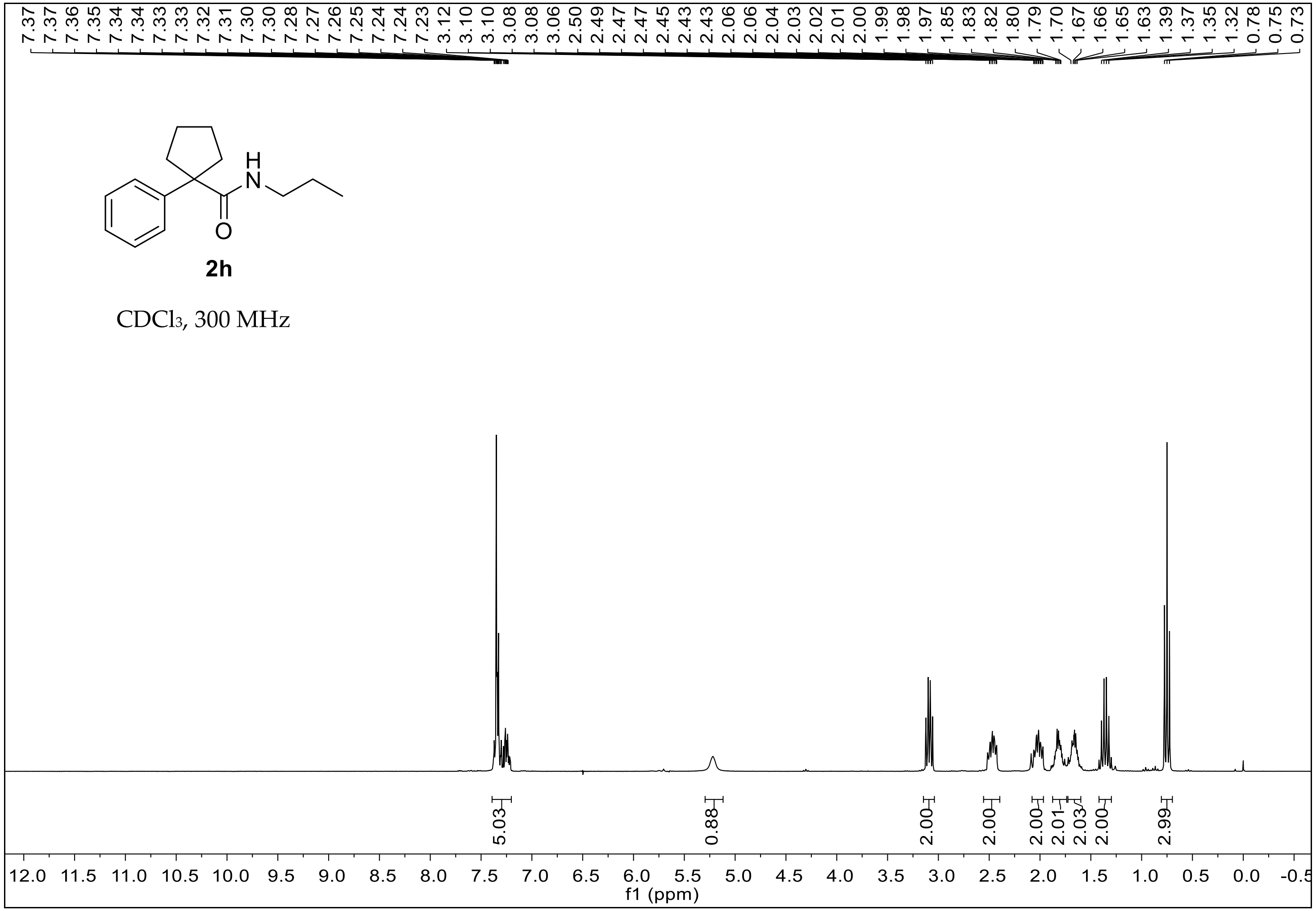

Figure S15. ^{1}H-NMR of 1-Phenyl-*N*-propylcyclopentane-1-carboxamide (**2h**).

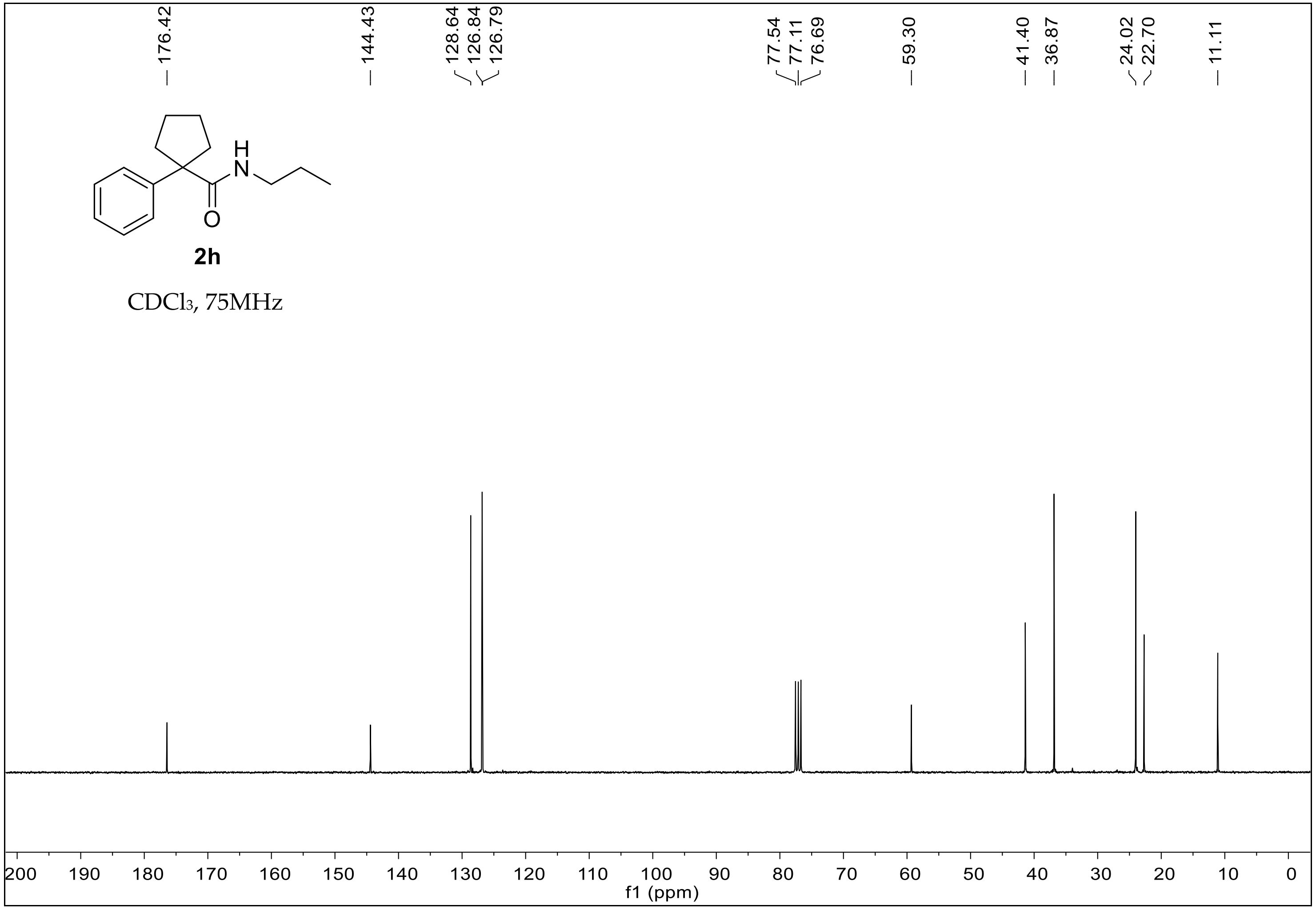

Figure S16. ^{13}C-NMR of 1-Phenyl-*N*-propylcyclopentane-1-carboxamide (**2h**).

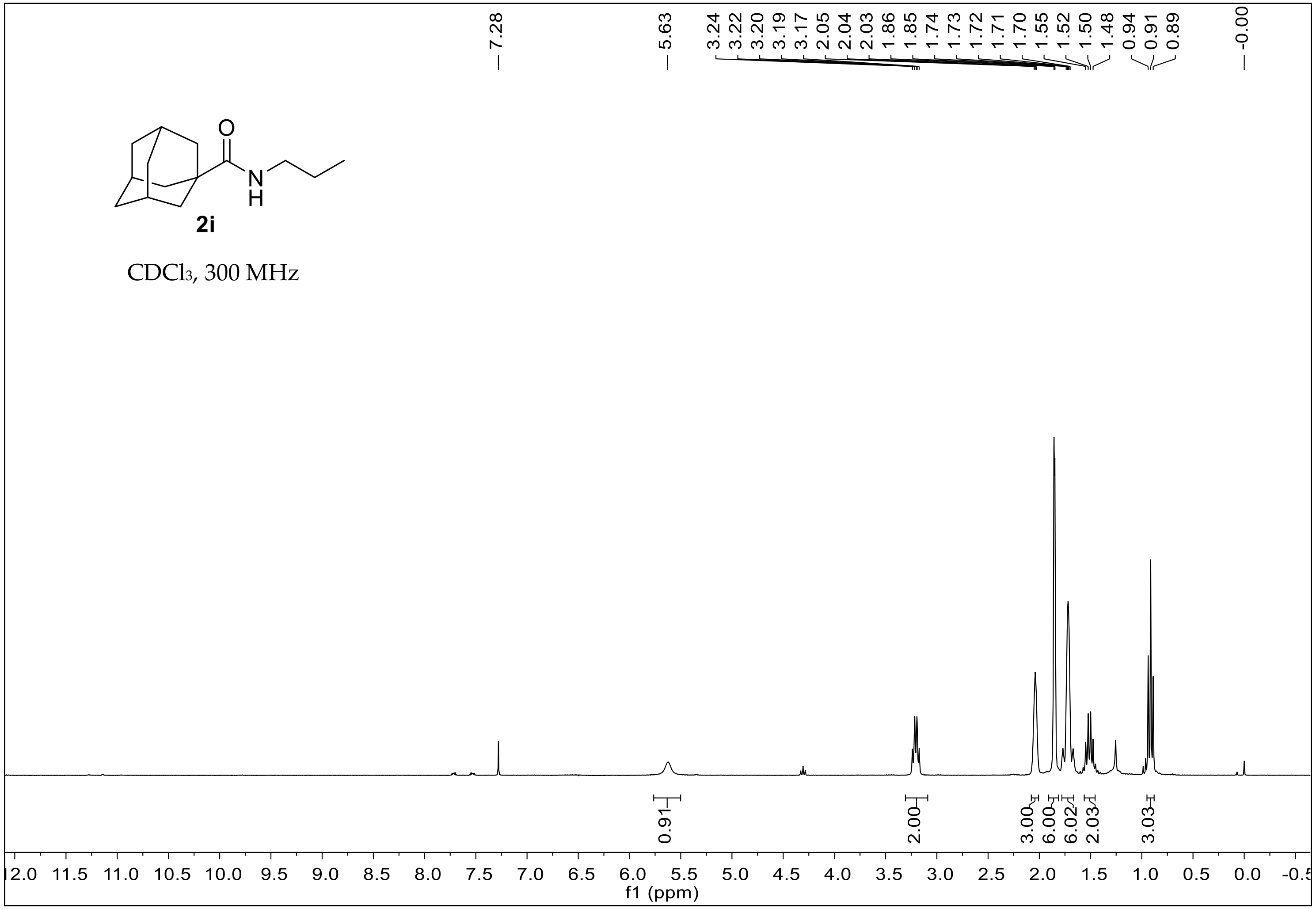

Figure S17. ^{1}H-NMR of (3r,5r,7r)-*N*-Propyladamantane-1-carboxamide (**2i**).

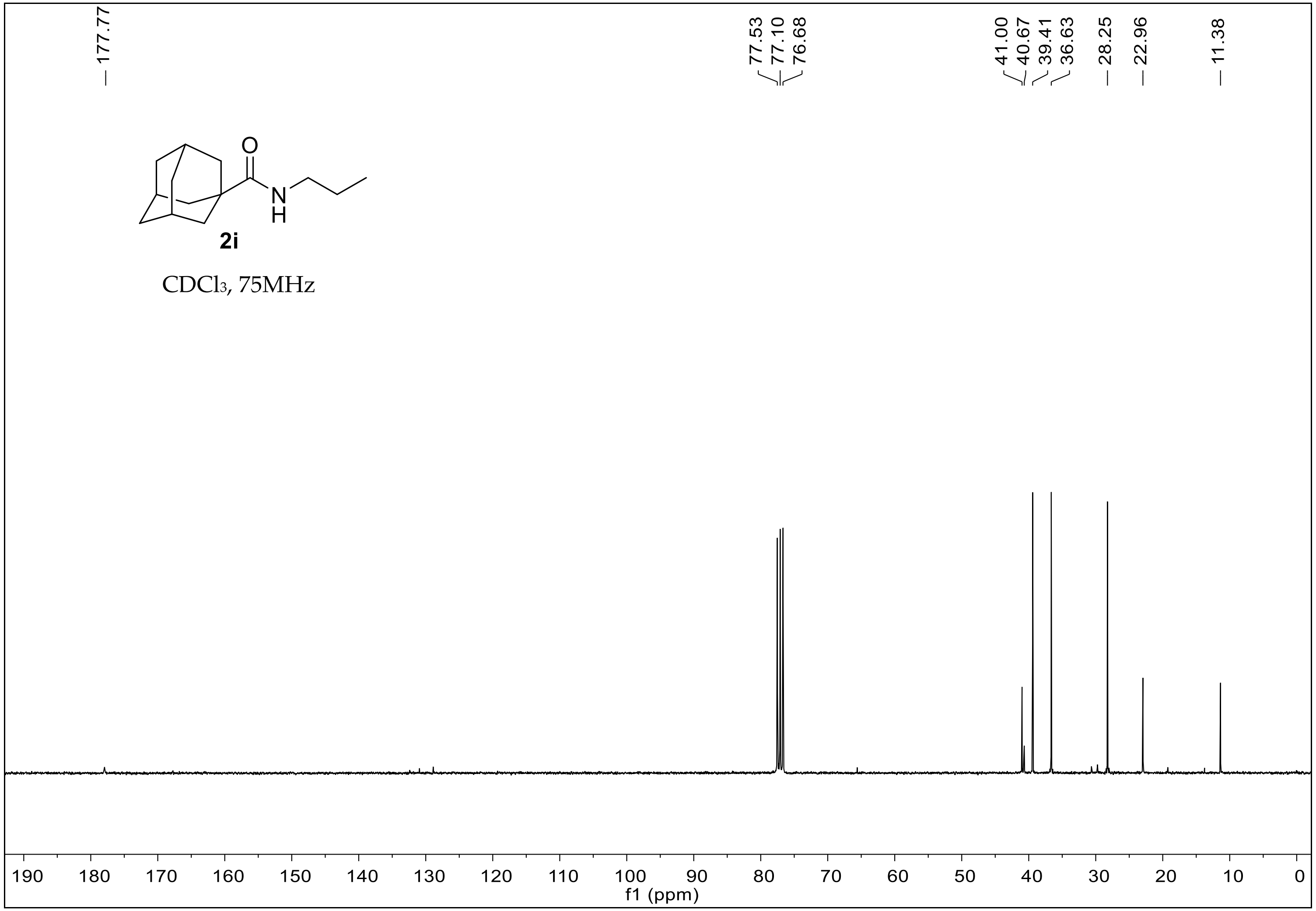

Figure S18. ^{13}C-NMR of (3r,5r,7r)-*N*-Propyladamantane-1-carboxamide (**2i**).

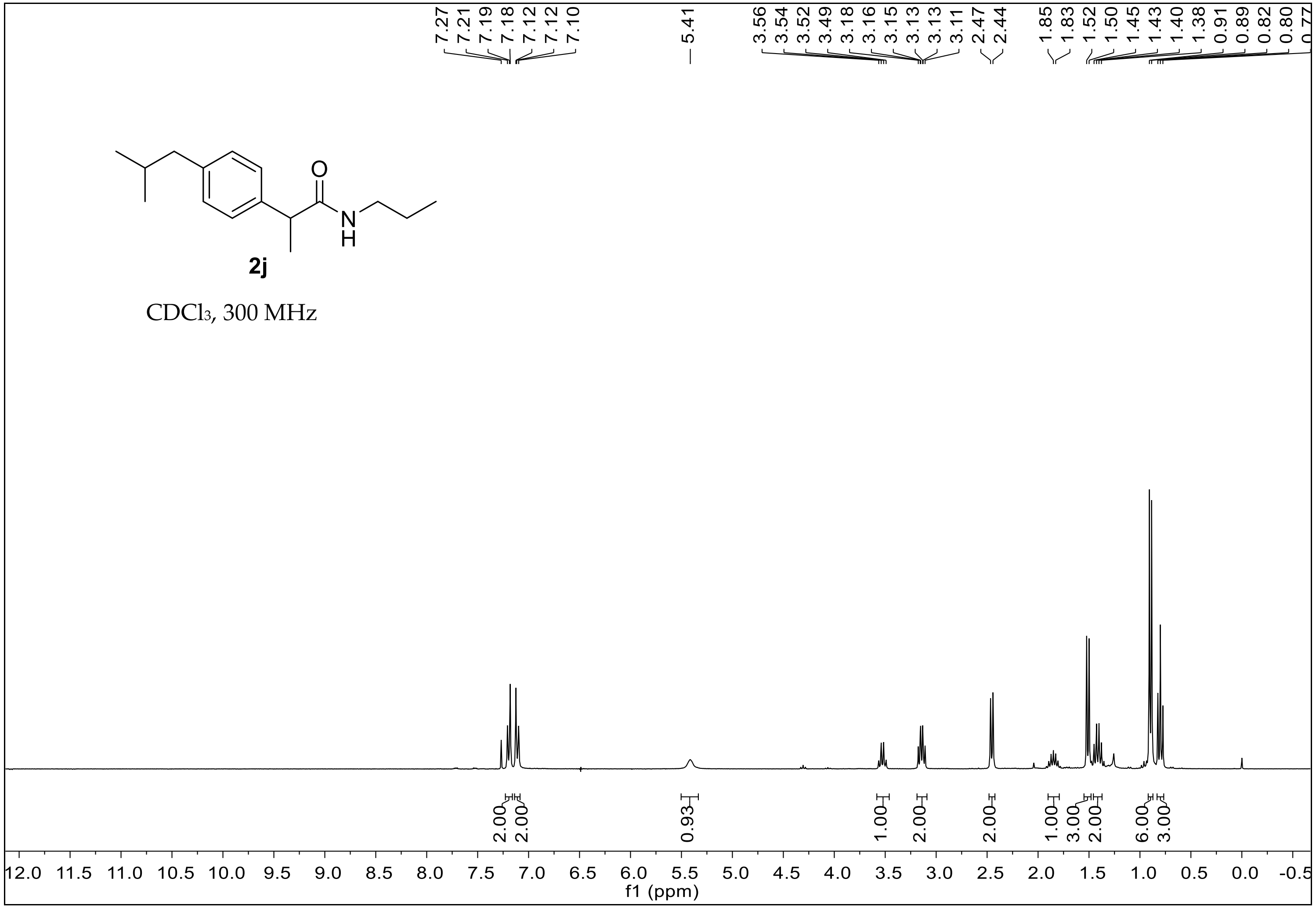

Figure S19. ^{1}H-NMR of 2-(4-Isobutylphenyl)-*N*-propylpropanamide (**2j**).

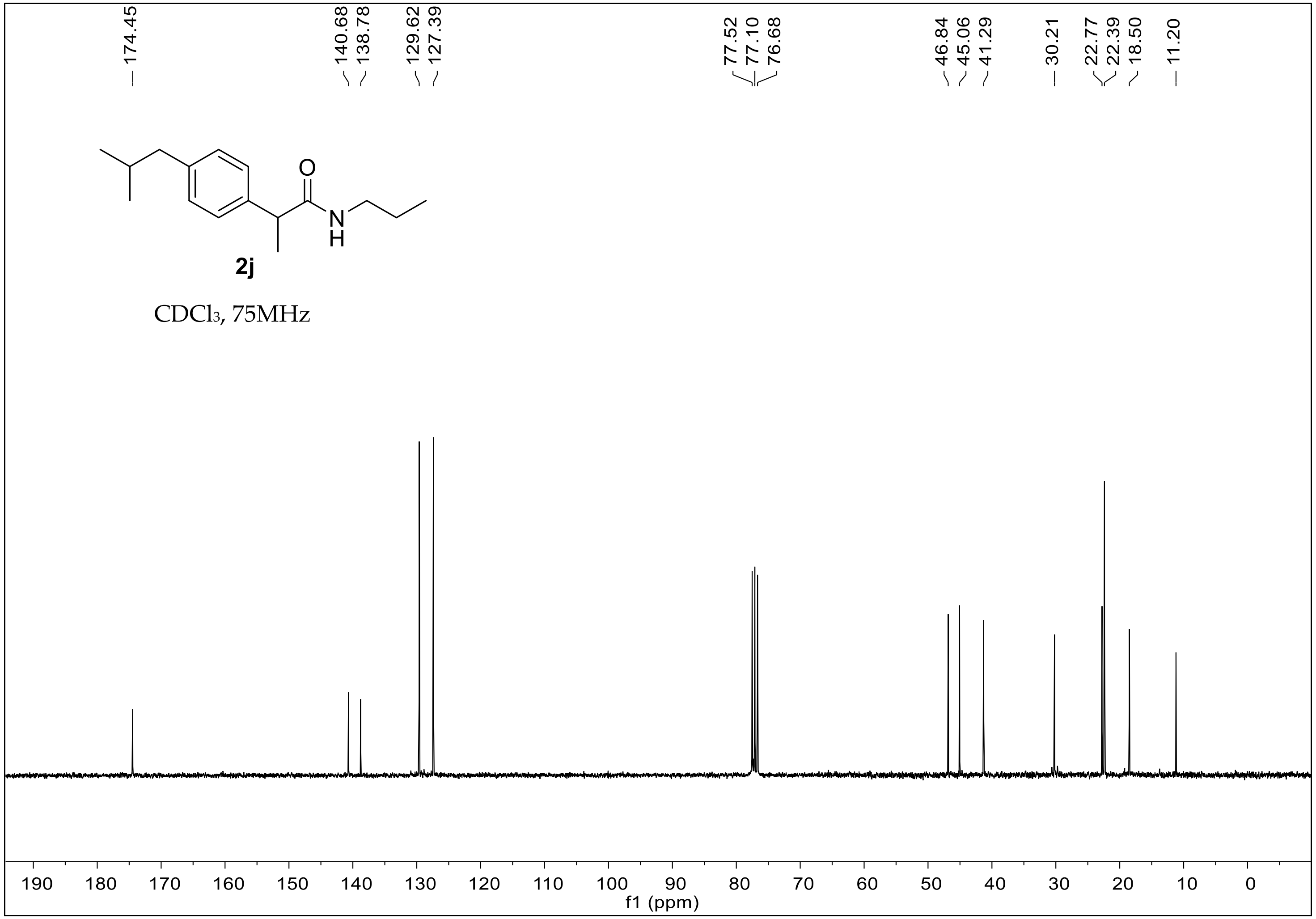

Figure S20. ^{13}C-NMR of 2-(4-Isobutylphenyl)-*N*-propylpropanamide (**2j**).

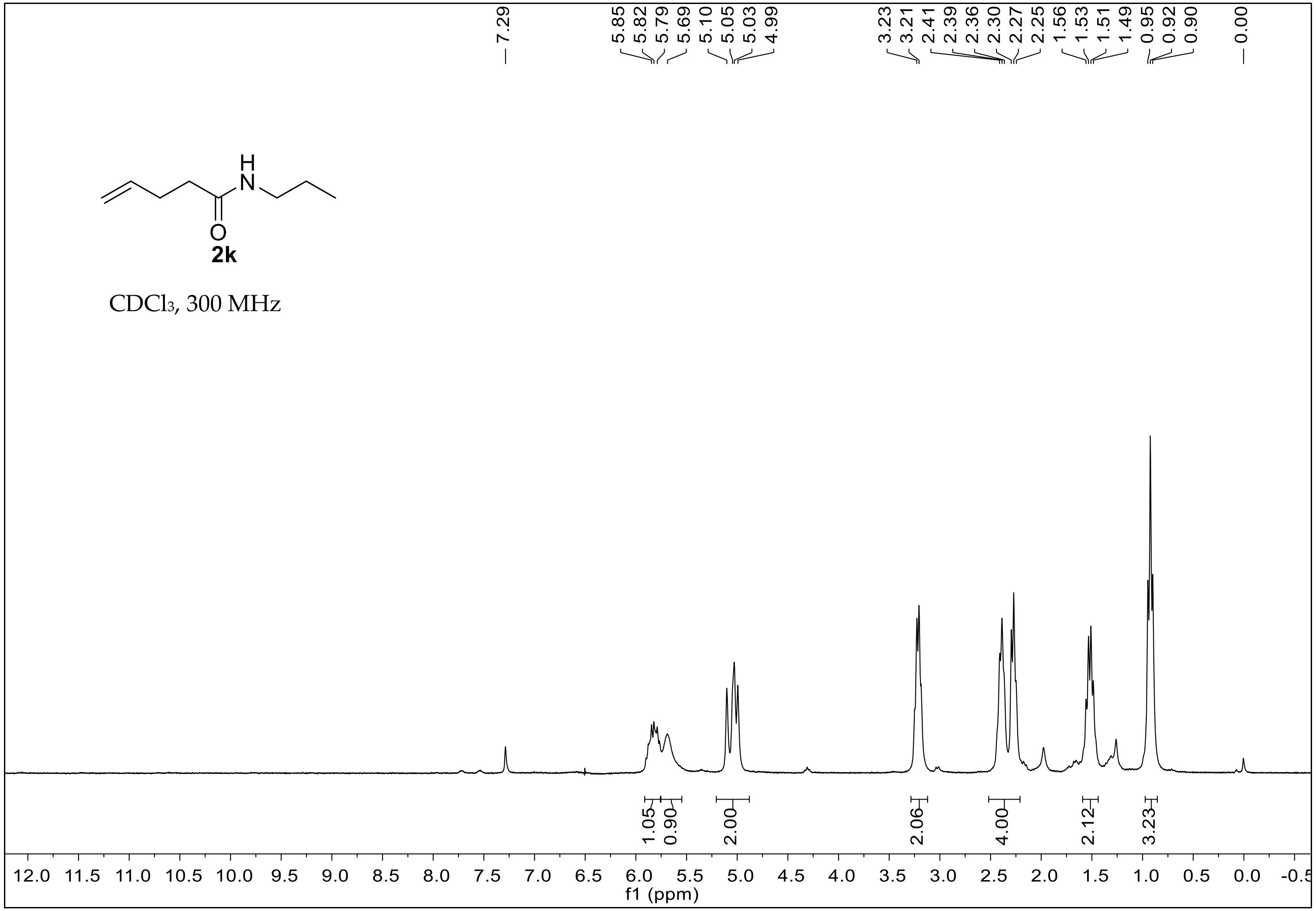

Figure S21. ^{1}H-NMR of *N*-Propylpent-4-enamide (**2k**).

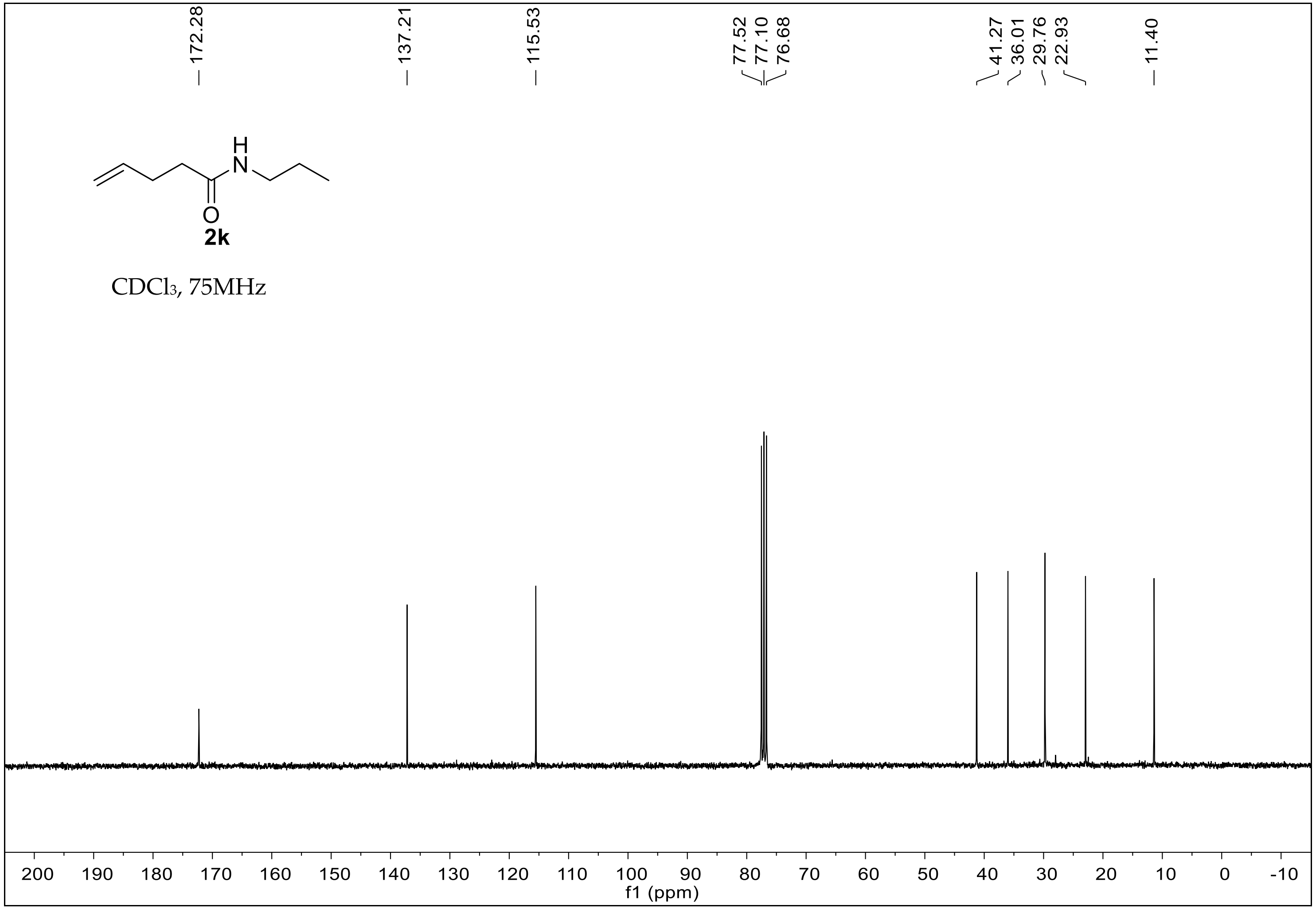

Figure S22. ^{13}C-NMR of *N*-Propylpent-4-enamide (**2k**).

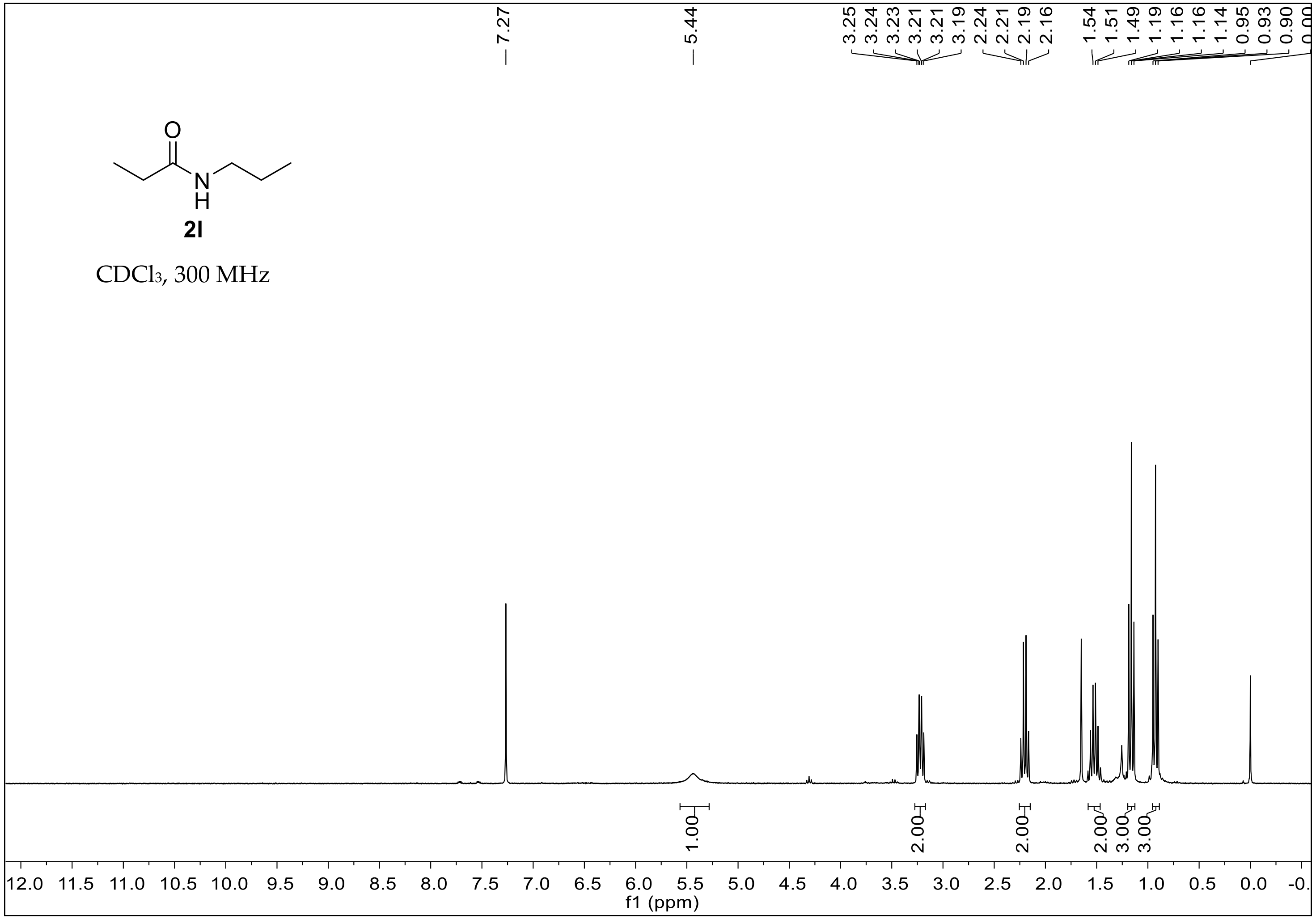

Figure S23. ¹H-NMR of *N*-Propylpropionamide (**2l**).

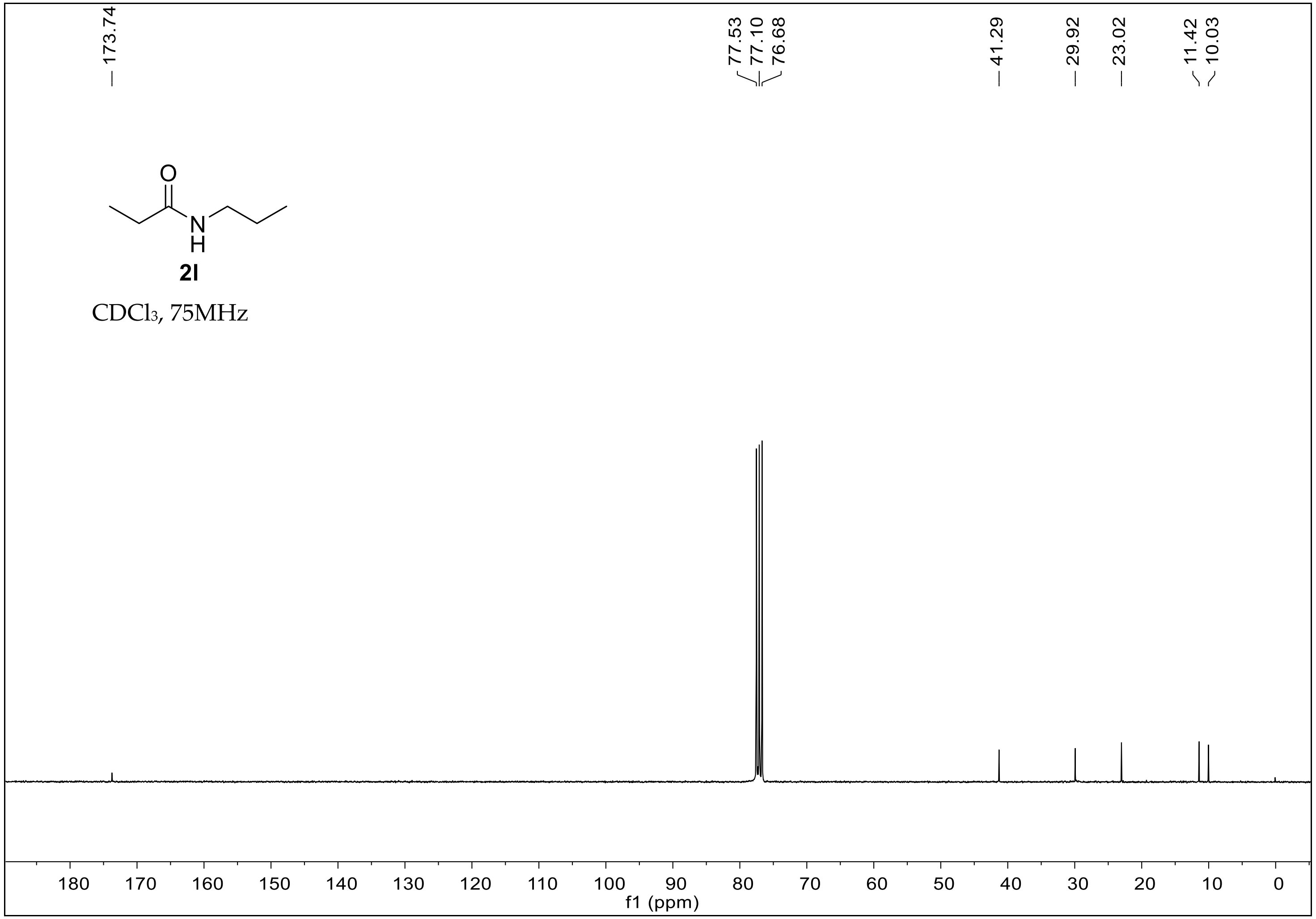

Figure S24. ^{13}C-NMR of *N*-Propylpropionamide (**2l**).

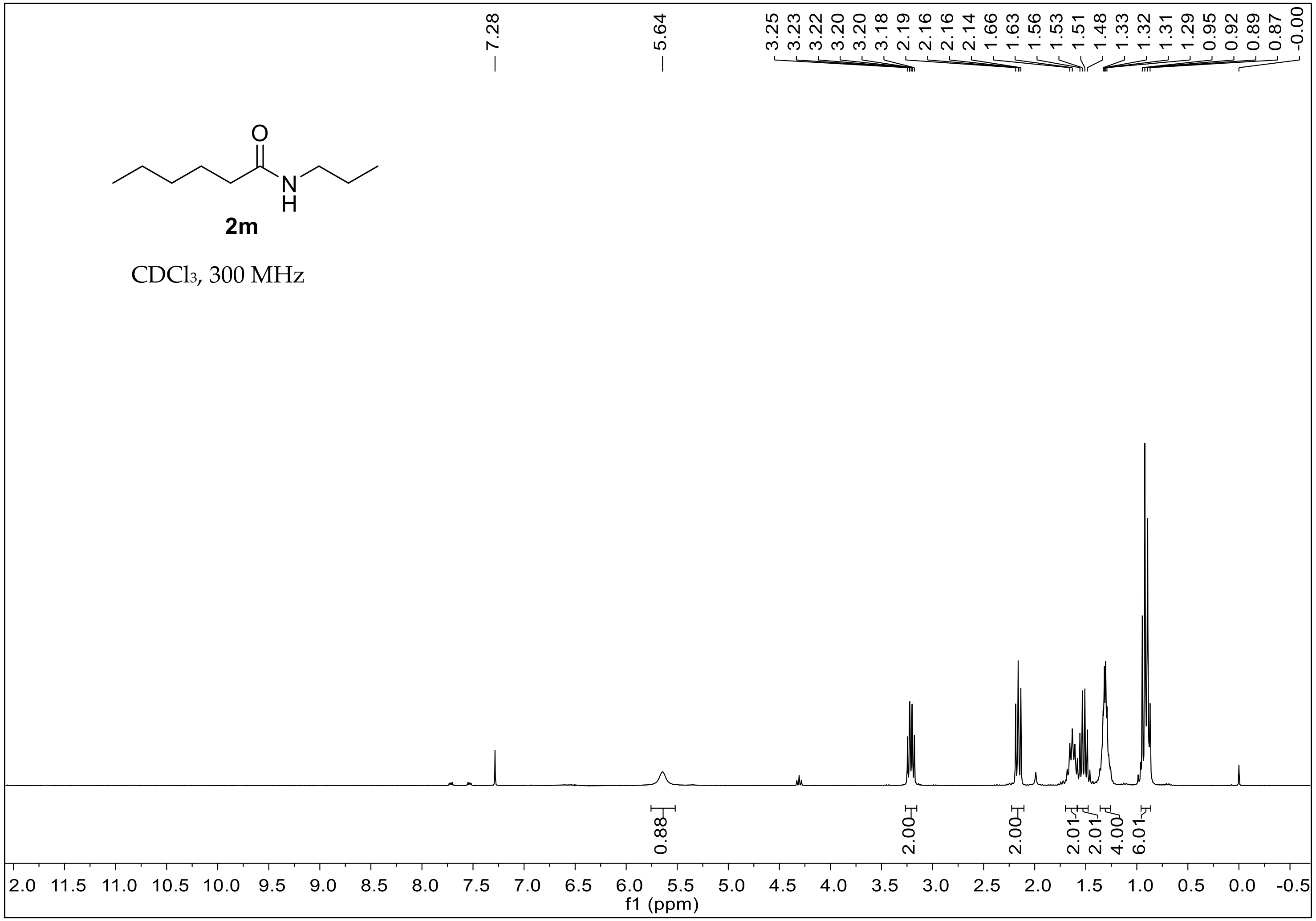

Figure S25. ^{1}H-NMR of *N*-Propylhexanamide (**2m**).

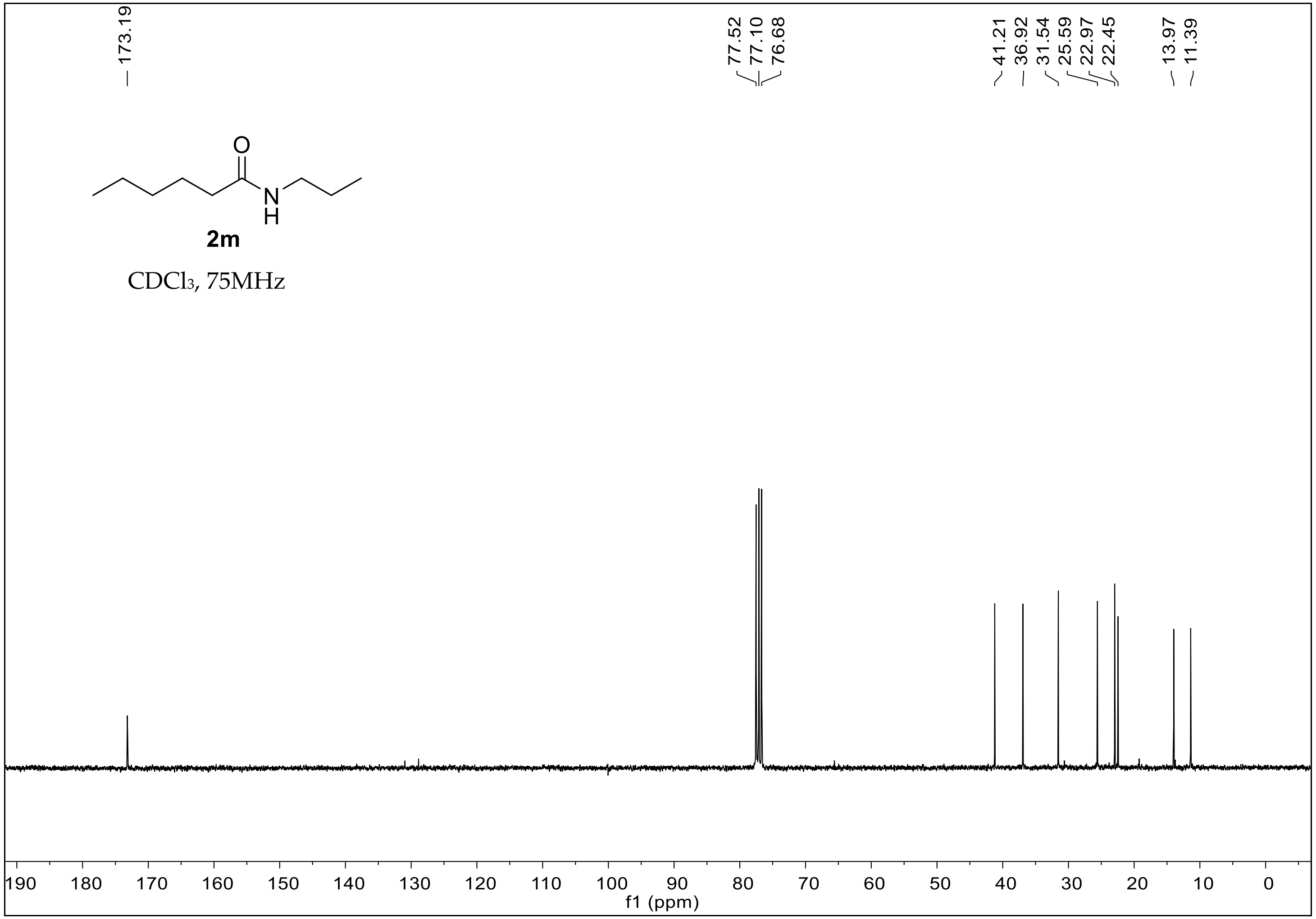

Figure S26. ^{13}C-NMR of *N*-Propylhexanamide (**2m**).

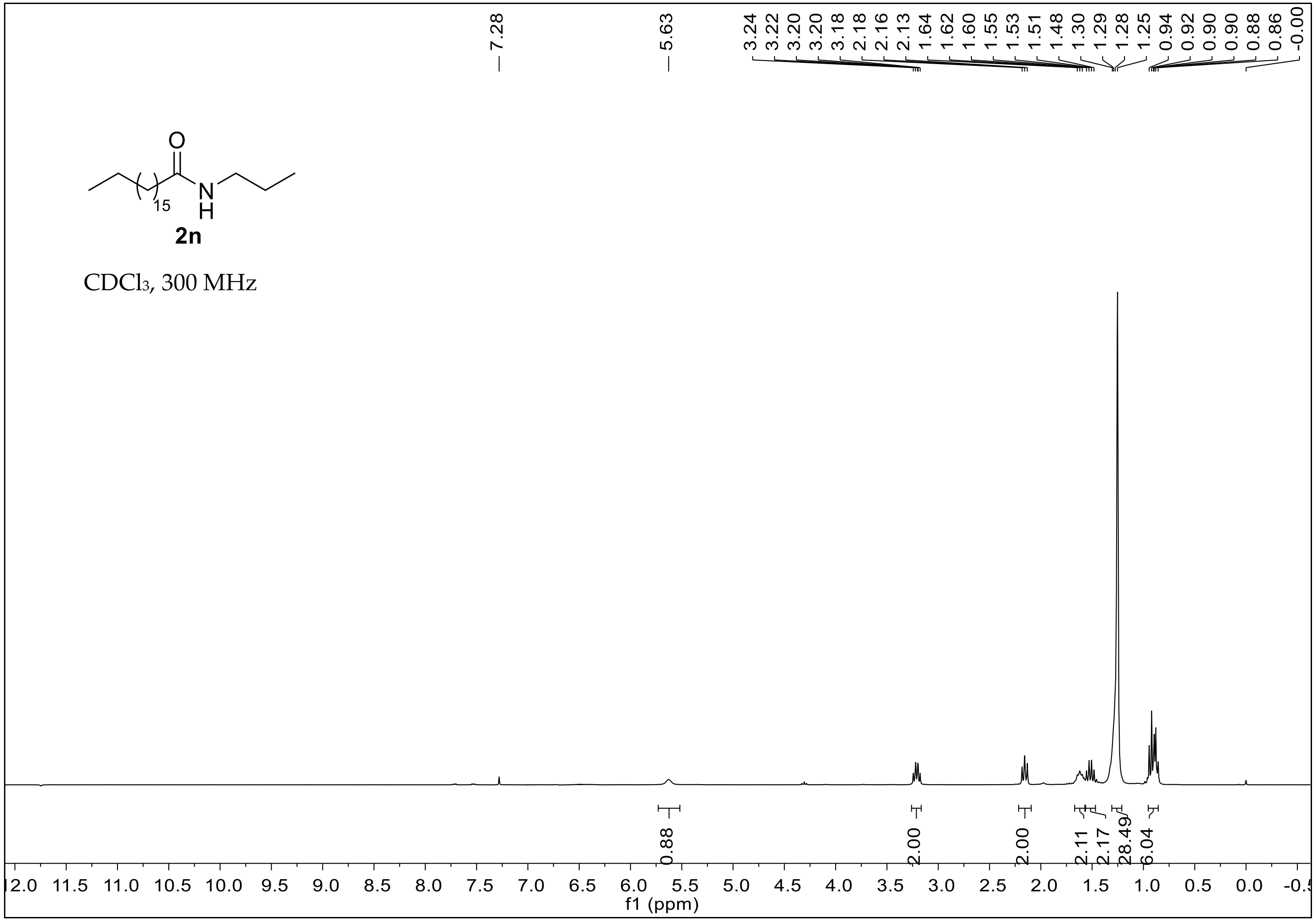

Figure S27. ^{1}H-NMR of *N*-Propylstearamide (**2n**).

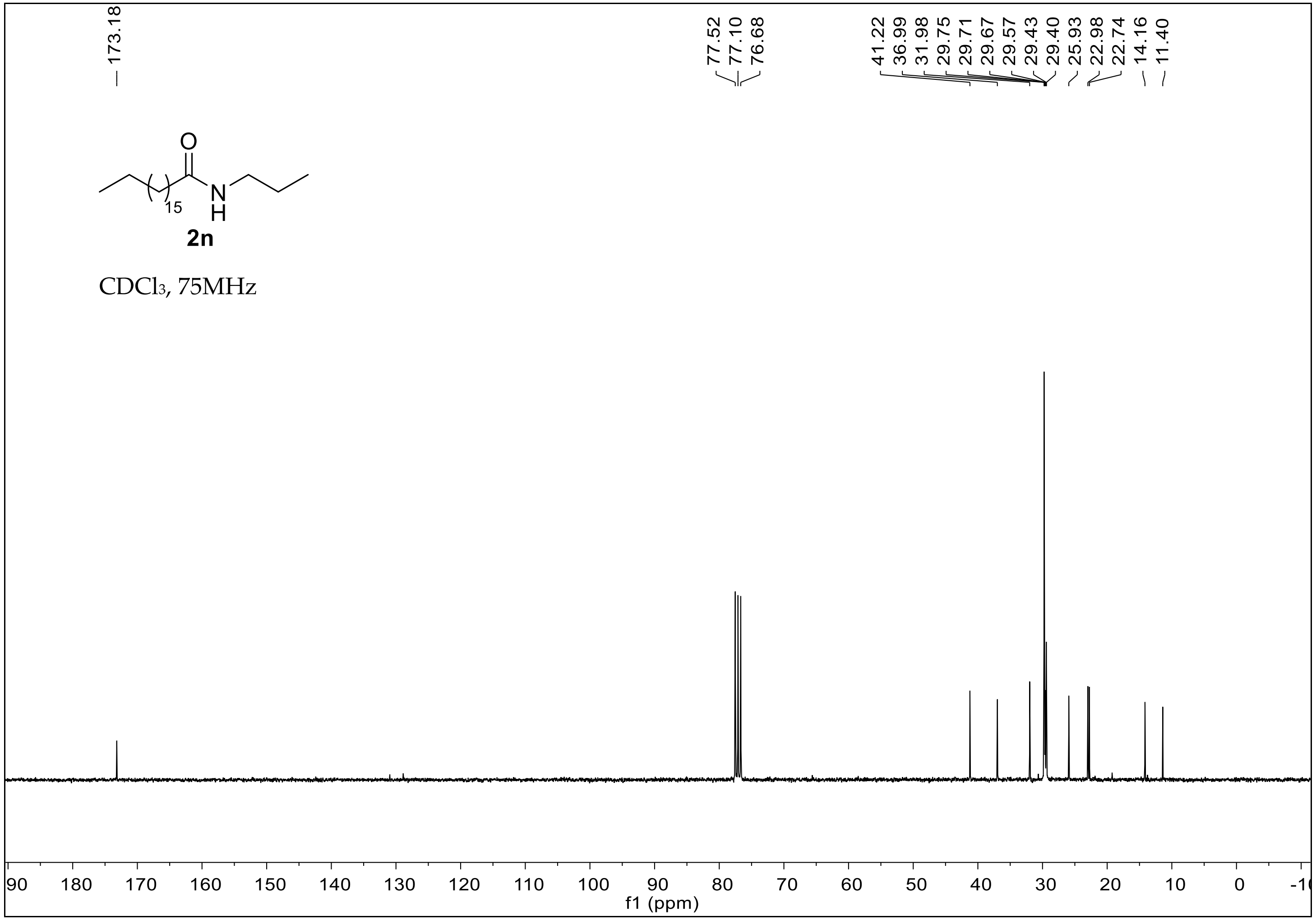

Figure S28. ^{13}C-NMR of *N*-Propylstearamide (**2n**).

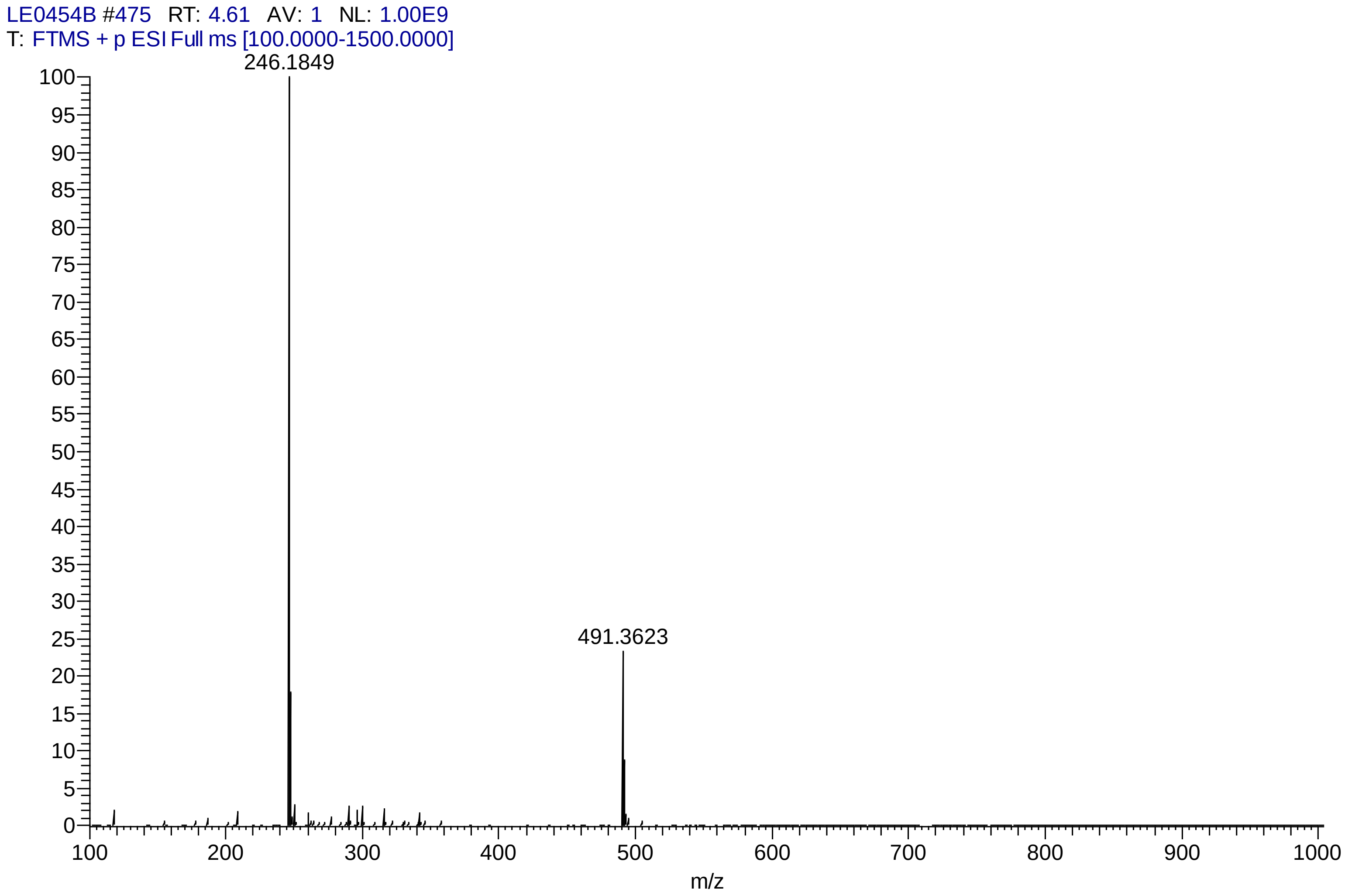

Figure S29. HRMS of 4-Cyclohexyl-*N*-propylbenzamide (**2g**).

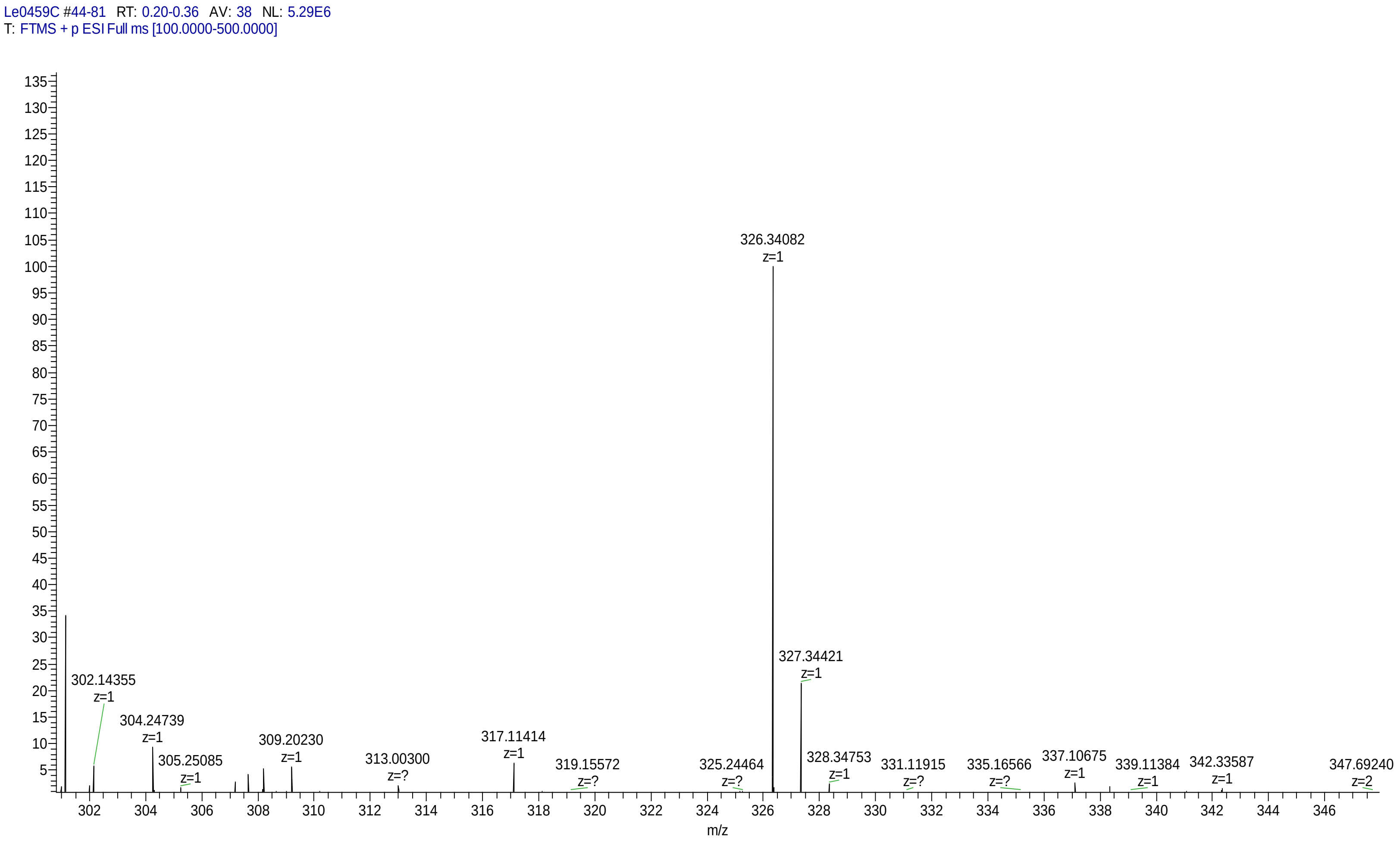

Figure S30. HRMS of *N*-Propylstearamide (**2n**).
